# A Preliminary *in vitro* and *in vivo* Evaluation of the Effect and Action Mechanism of 17-AAG Combined With Azoles Against Azole-Resistant *Candida* spp.

**DOI:** 10.3389/fmicb.2022.825745

**Published:** 2022-07-07

**Authors:** Luyao Liu, Xueying Zhang, Shruti Kayastha, Lihua Tan, Heng Zhang, Jingwen Tan, Linyun Li, Jinghua Mao, Yi Sun

**Affiliations:** ^1^Yangtze University, Jingzhou, China; ^2^Department of Medical Mycology, Shanghai Skin Disease Hospital, Tongji University School of Medicine, Shanghai, China; ^3^Clinical Lab, Jingzhou Hospital, Yangtze University, Jingzhou, China; ^4^Department of Cardiology, Jingzhou Hospital, Yangtze University, Jingzhou, China; ^5^Department of Dermatology, Jingzhou Hospital, Yangtze University, Candidate Branch of National Clinical Research Center for Skin and Immune Diseases, Jingzhou, China

**Keywords:** 17-AAG, *Candida auris*, azole-resistant *Candida*, synergy, Hsp90 inhibitor

## Abstract

Invasive candidiasis is the primary reason for the increased cases of mortality in a medical environment. The resistance spectra of *Candida* species to antifungal drugs have gradually expanded. Particularly, the resistance spectra of *Candida auris* are the most prominent. Hsp90 plays a protective role in the stress response of fungi and facilitates their virulence. In contrast, Hsp90 inhibitors can improve the resistance of fungi to antifungal drugs by regulating the heat resistance of Hsp90, which destroys the integrity of the fungal cell walls. Hsp90 inhibitors thus offer a great potential to reduce or address fungal drug resistance. The drugs tested for the resistance include itraconazole, voriconazole, posaconazole, fluconazole, and 17-AAG. A total of 20 clinical strains of *Candida* were investigated. The broth microdilution checkerboard technique, as adapted from the CLSI M27-A4 method, was applied in this study. We found that 17-AAG alone exerted limited antifungal activity against all tested strains. The MIC range of 17-AAG was 8 to >32 μg/ml. A synergy was observed among 17-AAG and itraconazole, voriconazole, and posaconazole against 10 (50%), 7 (35%), and 13 (65%) of all isolates, respectively. Moreover, the synergy between 17-AAG and fluconazole was observed against 5 (50%) strains of azole-resistant *Candida*. However, no antagonism was recorded overall. Our result adequately verifies the influence of 17-AAG on the formation of *Candida* spp. biofilm. Moreover, we determined that with the use of rhodamine 6G to detect drug efflux and that of dihydrorhodamine-123 to detect intracellular reactive oxygen species (ROS), treatment with 17-AAG combined with azole drugs could inhibit the efflux pump of fungi and promote the accumulation of ROS in the fungal cells, thereby inducing fungal cell apoptosis. Thus, the mechanism of 17-AAG combined with azoles can kill fungi. Our results thus provide a new idea to further explore drugs against drug-resistant *Candida* spp.

## Introduction

*Candida* is a symbiont of the normal skin and intestinal microbiota, inhabiting 30–70% of healthy individuals without causing any disease (Pappas et al., 2018). Invasive candidiasis (IC) is caused by increased fungal load and the rupture of the skin and mucosal membrane, which promotes fungal translocation or transmission into the bloodstream or cause damage to the host immune function. The mortality rate after *Candida* infection can be as high as 30–60% (Bugli et al., 2013; Arendrup and Patterson, 2017). In recent years, in parallel with the increasing use of endovascular prosthesis devices for heart diseases, the number of *Candida* patients with endocarditis prosthesis valve has also increased. The implantation of prosthetic valves and the subsequent biofilm formation in these patients has led to the development of resistance to antifungal drugs in them (Venditti, 2009). Clinical and *in vitro* resistance of *Candida* to commonly used azoles has significantly increased at present, especially that of *Candida auris* (Pristov and Ghannoum, 2019). As a type of iatrogenic pathogen, *C. auris* is widely distributed and considered highly contagious worldwide (Gonzalez-Lara and Ostrosky-Zeichner, 2020). In 2009, *C. auris* was isolated from the external auditory canal of a woman in a Tokyo hospital. However, as early as 1996, a Korean girl was reported to be infected with it (ElBaradei, 2020). Since then, *C. auris* infection has been reported throughout the world, excluding Antarctica (ElBaradei, 2020). Across the historical timeline, several outbreaks of *C. auris* infection have been reported across different parts of the world (ElBaradei, 2020). In addition, *C. auris* has reportedly demonstrated strong multi-drug resistance (MDR) and extensive drug resistance (XDR; Arendrup and Patterson, 2017). Several reports have provided evidence that most isolates (93%) are resistant to FLU, 35% to amphotericin B (AMB), and 7% to echinocandin. Echinocandins and AMB can be used as a drug for infection by *C. auris* (Bugli et al., 2013; Arendrup and Patterson, 2017; Pappas et al., 2018; Aruanno et al., 2019; ElBaradei, 2020); however, the cost of echinocandin and AMB substitutes is relatively higher. Specifically, in countries with limited resources, it is not easily available. Moreover, AMB has proven side-effects (Bugli et al., 2013; Arendrup and Patterson, 2017; Pappas et al., 2018; Aruanno et al., 2019; ElBaradei, 2020). Presently, there are only limited options of antifungal agents that can be used for the treatment of this infection. Cases of fungal resistance is increasing in parallel with the cost of developing new antifungal agents, especially in the past decades. Hence, there is an urgent need for establishing new combination therapy in this direction.

Heat shock proteins 90 (Hsp90), a group of highly conserved chaperones, stabilizes several proteins involved in basic metabolic reactions, accounting for 1–2% of all cytosolic proteins (McClellan et al., 2007; Scieglinska et al., 2019; Crunden and Diezmann, 2021). In the fungal stress response, Hsp90 protects fungi by activating important signaling pathways; this mechanism is considered beneficial for fungi to fungal volatilization (Mellatyar et al., 2018). Adenosine triphosphate is the main functional region of Hsp90 (Lamoth et al., 2012; Mellatyar et al., 2018; Aruanno et al., 2019), as has been confirmed with the use of internal and external models of *C. albicans* and *Cryptococcus* (McClellan et al., 2007; Refos et al., 2017; Aruanno et al., 2019). For instance, in *C. albicans* models, Hsp90 also controls temperature-dependent morphogenesis (Kim et al., 2019), while Hsp90 inhibitors can reduce drug resistance of fungi by destroying the integrity of fungal cell walls, inhibiting the transformation of yeast morphology to sporephore, and inhibiting the activity of ATPase at different temperatures (Lamoth et al., 2012; Aruanno et al., 2019; Li et al., 2020b). Therefore, the use of Hsp90 inhibitors combined with the existing antifungal agents for the treatment of fungal infection has broad prospects.

Geldanamycin—a member of the natural benzoquinone ansamycins family—is currently the most widely used Hsp90 inhibitor (Mellatyar et al., 2018). A synthetic variant of geldanamycin, 17-Allylamino-17-demethoxygeldamycin (17-AAG), exhibits similar biological activity and can competitively bind the ATP/ADP binding sites on Hsp90, thereby inhibiting its inherent ATPase activity (Lamoth et al., 2016; Li et al., 2020b) and reducing the resistance of fungi. Currently, it was applied at clinical stages II or III in multiple human tumor treatments and demonstrated satisfactory outcomes (McClellan et al., 2007). In the present study, we investigated the anti-*Candida* activity of 17-AAG, an Hsp90 inhibitor, alone or in combination with azoles under *in vitro* settings.

## Materials and Methods

### Fungal Isolates

A total of 20 clinical isolates of *Candida* spp., including 10 strains of *C. auris* and 10 strains of drug-resistant *Candida* were included in this study. All these strains were clinical isolates, and their identity was confirmed based on their microscopic morphology and molecular sequencing results (Tan et al., 2021). *C. auris* strains were obtained from the CDC and FDA Antibiotic Resistance Isolate Bank. *Candida parapsilosis* (ATCC22019) and *Candida krusei* (ATCC00279) was included in the study to ensure quality control.

### Antifungal Agents

All tested azoles, including ITR (No. S2476), VOR (No. S1442), POS (No. S1257), FLU (No. S1131), and 17-AAG (No. S1141), were bought in the powder form from Selleck Chemicals (China) and prepared in accordance with the Clinical and Laboratory Standards Institute’s (CLSI) broth microdilution method M27-A4 (Blum et al., 2013) The working concentration range of 17-AAG was 0.25–32 μg/ml, while those of ITR, VOR, and POS were 0.125–16 μg/ml for *C. auris*. For drug-resistant *Candida spp*., the working concentration ranges of ITR, VOR, POS, and FLU were 0.06–8 μg/ml, 0.06–8 μg/ml, 0.03–4 μg/ml, and 0.25–32 μg/ml.

### Inoculum Preparation

Conidia were freshly collected in sterile distilled water from cultures grown for 2 days on Sabouraud’s dextrose agar (SDA) and then diluted to the right concentration of 1–5 × 10^6^ cfu/ml. Then, the suspensions were further diluted 1,000 times in the RPMI-1640 medium to achieve the desired density or approximately 1–5 × 10^3^ cfu/ml concentration.

### Testing the *in vitro* Synergy of 17-AAG and Azoles

The combination groups with 17-AAG and azoles against all test strains were estimated through broth microdilution checkerboard technique, which was performed in accordance with the CLSI M27-A4 standards. Briefly, 50 μl of serially diluted 17-AAG and another 50 μl of serially diluted azoles were inoculated on a 96-well plate in the horizontal and vertical directions, separately. Finally, the prepared plates were inoculated with 100 μl of the prepared fungal suspension and incubated for 24 h at 35°C, followed by the interpretation of the observation. The minimum inhibitory concentrations (MICs) were visually determined by the inhibition of 50% in the control group, as established by the standardized endpoint (Blum et al., 2013). The fractional inhibitory concentration index (FICI) was applied to analyze the interactions between 17-AAG and azoles (Odds, 2003), which was calculated by the following formula: FICI = d1/(D1)p + d2/(D2)p, where d1 and d2 are the doses of tested agents in combination, while (D1)p and (D2)p are the doses of two respective compounds alone. If an interaction was scored as FICI of ≤0.5, synergy; as FICI of >0.5 to ≤4, indifference and as FICI of >4, antagonism (Van Dijck et al., 2018). All experiments were conducted in triplicate.

### Evacuation of *in vivo* Drug Sensitivity of 17-AAG Alone and in Combination With Azoles

Further to the abovementioned results, we selected the sixth instar (300 mg; Sichuan, China) to evaluate the *in vivo* interaction between 17-AAG and azoles referred in our previous study (Tan et al., 2021). Accordingly, *C. auris* AR 382*, C. abicans* R2, and *C. glabrata* 05448 were tested. Before the experiment, fresh spore suspension was prepared at the concentration of 1 × 10^8^ cfu/ml. Then, 17-AAG was diluted with azoles. The solution in the monotherapy group was diluted to 200 mg/ml with normal saline, while that in the combination group was mixed in advance and diluted to 200 mg/ml with normal saline. The larvae were randomly categorized into 11 groups, as follows: untreated, normal saline, Conidal, POS, ITR, VOR, FLU, 17-AAG + POS, 17-AAG + ITR, 17-AAG + VOR, and 17-AAG + FLU groups. Then, 10 μl of the corresponding spore suspension was injected using a Hamilton syringe (25 gauge, 50 μl) from the last left foot of each set of larvae, 5 μl (1 μg/worm) was injected at 2-h apart, and the larvae were cultured at 37°C, within 120 h of injection. The survival of the larvae was monitored every 24 h. The larval survival curve was evaluated by Kaplan–Meier method and log-rank (Mantel-Cox) test, with *p* < 0.05 set as a significance threshold.

### Biofilm Drug Sensitivity Testing

Sabouraud dextrose broth (SAB; 2 ml) was added to each well of a 24-well plate, to which fresh suspension of *C. auris* AR 382 was added at the final concentration of 1 × 10^6^ cfu/ml. Then, a predetermined concentration of the drug was added. The concentration of the drug was based on the experimental results of the broth microdilution checkerboard technique (Table 1). We divided the experiment into 8 groups, as follows: No drug,17-AAG (16 μg/ml), ITR (0.5 μg/ml), VOR (0.5 μg/ml), POS (1 μg/ml), 17-AAG + ITR (0.25 μg/ml + 0.125 μg/ml), 17-AAG + VOR (8 μg/ml + 0.125 μg/ml), and 17-AAG + POS (4 μg/ml + 0.125 μg/ml) groups. Finally, after incubation at 121°C for 30 min, autoclaved round slices were added to a 24-well plate, followed by their incubation at 37°C for 24–48 h. Two experimental subgroups were created for each group. At the time point of 24 h and 48 h, the round slices was removed with tweezers and placed on the slide. A drop of fluorescent staining solution Calcofluor White stain (Sigma 18,909) and another drop of 10% KOH were added to the slide, followed by the observation of biofilm under fluorescence microscope. In order to further evaluate the effect of different antifungal agents on *C. auris* AR 382, the spores above the round slices were re-suspended after observation of the biofilm at 24 h and 48 h. The CFU were counted for different groups and evaluated by paired *T* test. The experiment was repeated thrice.

**Table 1:** MICs and FICIs results with the combinations of 17-AAG and azoles against azole-resistant *Candida* spp.

Strains	Species	MIC^1^ (μg/m) for							
		Agent alone				Combination^2^		
		17-AAG	ITR	VOR	POS	17-AAG/ITR	17-AAG/VOR	17-AAG/POS
AR381	*C. auris*	>32	0.5	0.125	0.125	2/0.25(0.53)	0.5/0.125(1.01)	0.5/0.125(1.01)
AR382		16	0.5	0.5	1	0.5/0.125(0.28)	8/0.125(0.75)	4/0.125(0.38)
AR383		>32	0.25	2	0.5	1/0.125(0.52)	0.5/0.125(0.07)	2/0.125(0.28)
AR384		>32	1	0.5	0.25	0.5/1(1.01)	0.5/0.5(1.01)	0.5/0.125(0.51)
AR385		>32	0.5	16	1	0.5/0.5(1.01)	16/8(0.75)	0.5/0.125(0.13)
AR386		16	0.5	16	0.5	0.5/0.125(0.28)	16/16(2)	2/0.125(0.38)
AR387		16	0.25	1	1	0.5/0.25(1.03)	0.5/0.125(0.16)	0.5/0.125(0.16)
AR388		>32	1	2	0.125	0.5/1(1.01)	0.5/1(0.51)	0.5/0.125(1.01)
AR389		8	0.25	4	0.25	0.5/0.125(0.56)	0.5/2(0.56)	1/0.25(1.13)
AR390		>32	1	4	0.5	1/0.125(0.14)	1/1(0.27)	1/0.125(0.27)
64,550	*C. albicans*	>32	2	0.25	2	32/0.25(0.63)	0.5/0.125(0.51)	16/0.25(0.38)
5,310		16	2	0.125	0.06	0.5/0.125(0.09)	0.5/0.125(1.03)	2/0.03(0.63)
R2		16	1	0.5	0.06	4/0.125(0.38)	0.5/0.125(0.28)	0.5/0.03(0.53)
R9		16	16	16	8	2/0.125(0.13)	0.5/0.125(0.04)	1/0.03(0.07)
R14		16	4	8	1	1/0.125(0.09)	0.5/4(0.53)	8/0.5(1.00)
R15		32	1	1	1	1/0.125(0.16)	0.5/0.125(0.14)	4/0.03(0.16)
5,448	*C. glabrata*	>32	8	2	2	2/2(0.28)	4/2(1.06)	1/0.5(0.27)
5,150	*C. tropicalis*	>32	0.5	0.125	0.25	0.5/0.125(0.26)	0.5/0.125(1.01)	2/0.06(0.27)
ATCC22019	*C. parapsilosis*	>32	0.5	0.125	0.125	1/0.25(0.52)	0.5/0.125(1.01)	0.5/0.125(1.01)
ATCC00279	*C. krusei*	>32	0.25	1	1	0.5/0.125(0.51)	1/0.25(0.27)	8/0.25(0.38)

1 *The MIC is the concentration resulting in 50% growth inhibition*.

2 *Fractional inhibitory concentration index (FICI) results are shown in parentheses. Synergy (FICI < 0.5); no interaction (indifference, 0.5 < FICI < 4).*

### Testing the Extracellular Rhodamine 6G

Fresh bacterial suspension of *C. auris* AR 382 (1 × 10^9^ cfu/ml) was added to 6 ml of the 1× PBS solution. After twice centrifugation at 4,000 rpm, the solution was re-suspended with PBS and cultured for 2 h in a shaker. After centrifugation at 4,000 rpm, the intracellular energy was exhausted and the solution was re-suspended with 10 μm of PBS R6G (Sigma 10 μm) liquor, mixed uniformly, and placed at 37°C shaking at 200 rpm for 50 min, followed by incubation on an ice bath for 10 min and twice centrifugation at 4,000 rpm. Next, 6 ml of the PBS solution was added to the control without adding any drugs. In the sugar-free and drug-free control groups, only 6 ml of the PBS solution was added without any additional sugar, and the remaining drugs with predetermined concentration were added to each tube (Table 1) as follows: no drug, no glucose, 17-AAG (16 μg/ml), ITR (0.5 μg/ml), VOR (0.5 μg/ml), POS (1 μg/ml), 17-AAG + ITR (0.25 μg/ml + 0.125 μg/ml), 17-AAG + VOR (8 μg/ml + 0.125 μg/ml), and 17-AAG + POS (4 μg/ml + 0.125 μg/ml). The samples were assayed at 0 min, and glucose was added after 10 min. After each sampling, the supernatant was centrifuged and added to a 96-well plate. A spectrophotometer (Beijing Perlong DNM-9602; excitation light 527 nm) was used to measure the fluorescence in the supernatant due to efflux, once every 10 min, for 1 h. The experiment was repeated thrice on different days to evaluate the induction effect of drugs on the fungal efflux pump.

### Testing the Intracellular ROS

The fresh suspension of *C. auris* AR 382 was added to 10 ml of SAB at the final concentration of 3–5 × 10^6^ cfu/ml. Germs were treated with the azoles of predetermined concentration {No drug, 17-AAG (16 μg/ml), ITR (0.5 μg/ml), VOR (0.5 μg/ml), POS (1 μg/ml), 17-AAG + ITR (0.25 μg/ml + 0.125 μg/ml), 17-AAG + VOR (8 μg/ml + 0.125 μg/ml), and 17-AAG + POS (4 μg/ml + 0.125 μg/ml)} and dihydrorhodamine-123 (Sigma DHR-123 5 μg/ml), followed by incubation at 37°C for 1 h. After centrifugation and resuspension, the germlings were harvested and detected by flow cytometry (Beckman Coulter DxFLEX) at an excitation wavelength of 488–505 nm and an emission wavelength of 515–575 nm. The experiment was repeated thrice on different days, and *p* values were calculated using unpaired *T* test.

## Results

### 17-AAG and Azoles Interactions *in vitro*

**Figure 1:**
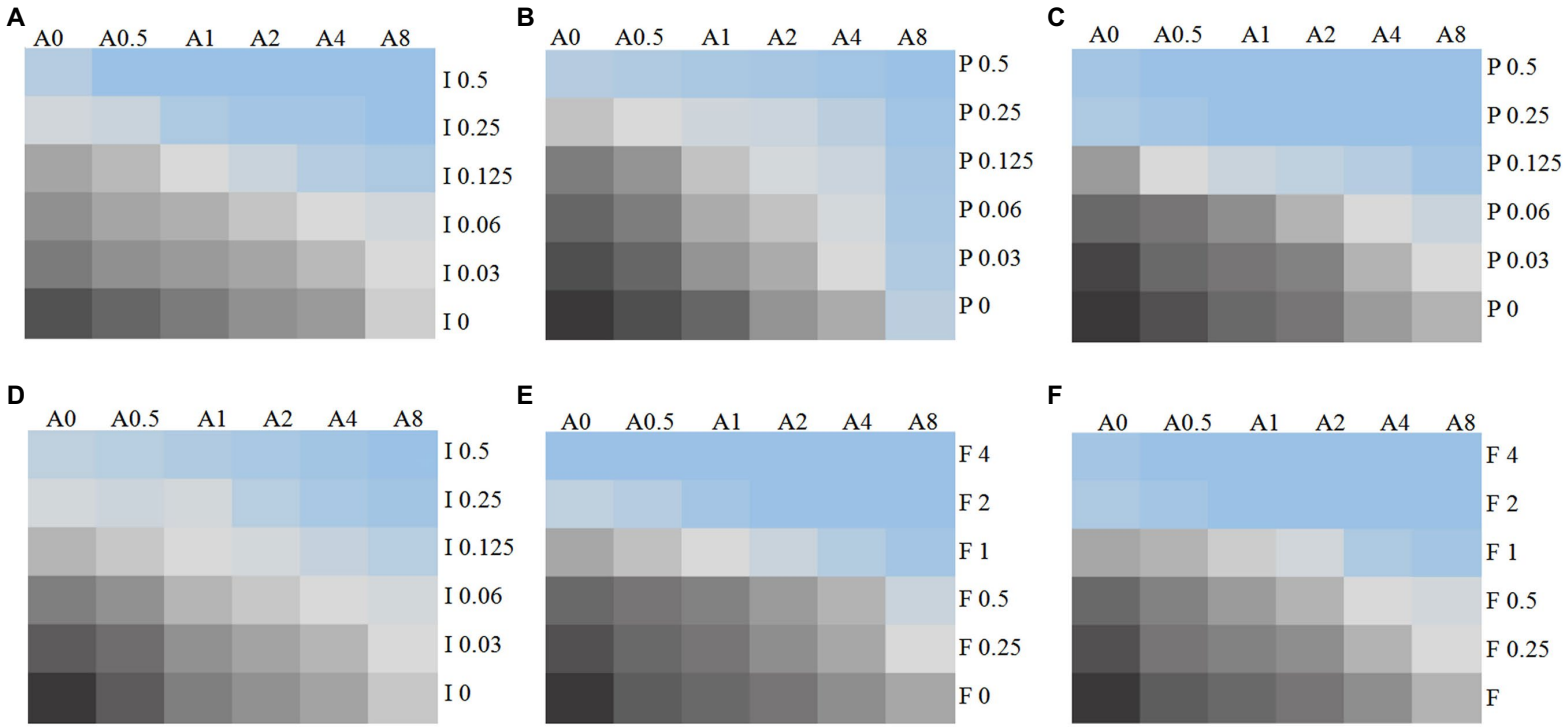
Chromatic scale diagram of synergistic effects of partial drug sensitivity results. Several drug sensitivity diagrams with obvious synergistic effect are listed in accordance with the *in vitro* drug sensitivity test and checkerboard method, **(A)** *C. auris* AR 382 17-AAG + ITR; **(B)** *C. auris* AR 382 17-AAG + POS; **(C)** 5,150 17-AAG + POS; **(D)** R9 ITR; **(E)** R2 FLU; and **(F)** 5,150 FLU. From black to blue, indicating that the fungus growth gradually decreased, A represents 17-AAG, I represents ITR, V represents VOR, P represents POS, and the number after the letter represents concentration (μg/ml).

The MICs ranges of each drug against all *Candida* isolates were 8 to >32 μg/ml for 17-AAG, 0.25–1 μg/ml for ITR, 0.125–4 μg/ml for VOR, 0.06–2 μg/ml for POS, and 0.5–64 μg/ml for FLU, respectively (Tables 1, 2). Moreover, 17-AAG alone exhibited only a slight antifungal activity against all tested strains. However, when 17-AAG was combined with ITR, VOR, or POS, synergistic anti-*Candida* effects were detected in 10 (50.0%), 7 (35.0%), and 13 (65.0%) isolates, while, in combination with FLU, the corresponding effect was observed in 5 (50%) isolates of drug-resistant *Candida* spp. The concentrations of 17-AAG in the synergistic combinations were effective within the range of 0.5–32 μg/ml (Tables 1, 2; Figure 1). No antagonism was recorded in the two combination groups.

**Table 2:** MICs and FICIs results with the combinations of 17-AAG and FLU against azole-resistant *Candida.*

Strains	Species	MIC (μg/ml)^1^ for		
		Agent alone		Combination^2^
		17-AAG	FLU	17-AAG/FLU
5310	*C. albicans*	16	0.5	0.25/0.5(1.02)
64550		>32	16	16/4(0.50)
R2		16	2	4/0.5(0.50)
R9		16	64	2/0.5(0.13)
R14		16	16	8/8 (1.00)
R15		32	16	4/1(0.19)
5448	*C. glabrata*	>32	64	0.5/64(1.01)
5150	*C. tropicalis*	>32	2	2/0.5(0.28)
ATCC22019	*C. parapsilosis*	>32	4	4/4(1.06)
ATCC00279	*C. krusei*	>32	64	2/32(0.53)

1 *The MIC is the concentration resulting in 50% growth inhibition*.

2 *Fractional inhibitory concentration index (FICI) results are shown in parentheses. Synergy (FICI < 0.5); no interaction (indifference, 0.5 < FICI < 4).*

### Evacuation of *in vivo* Drug Sensitivity of 17-AAG Alone and in Combination With Azoles

**Figure 2:**
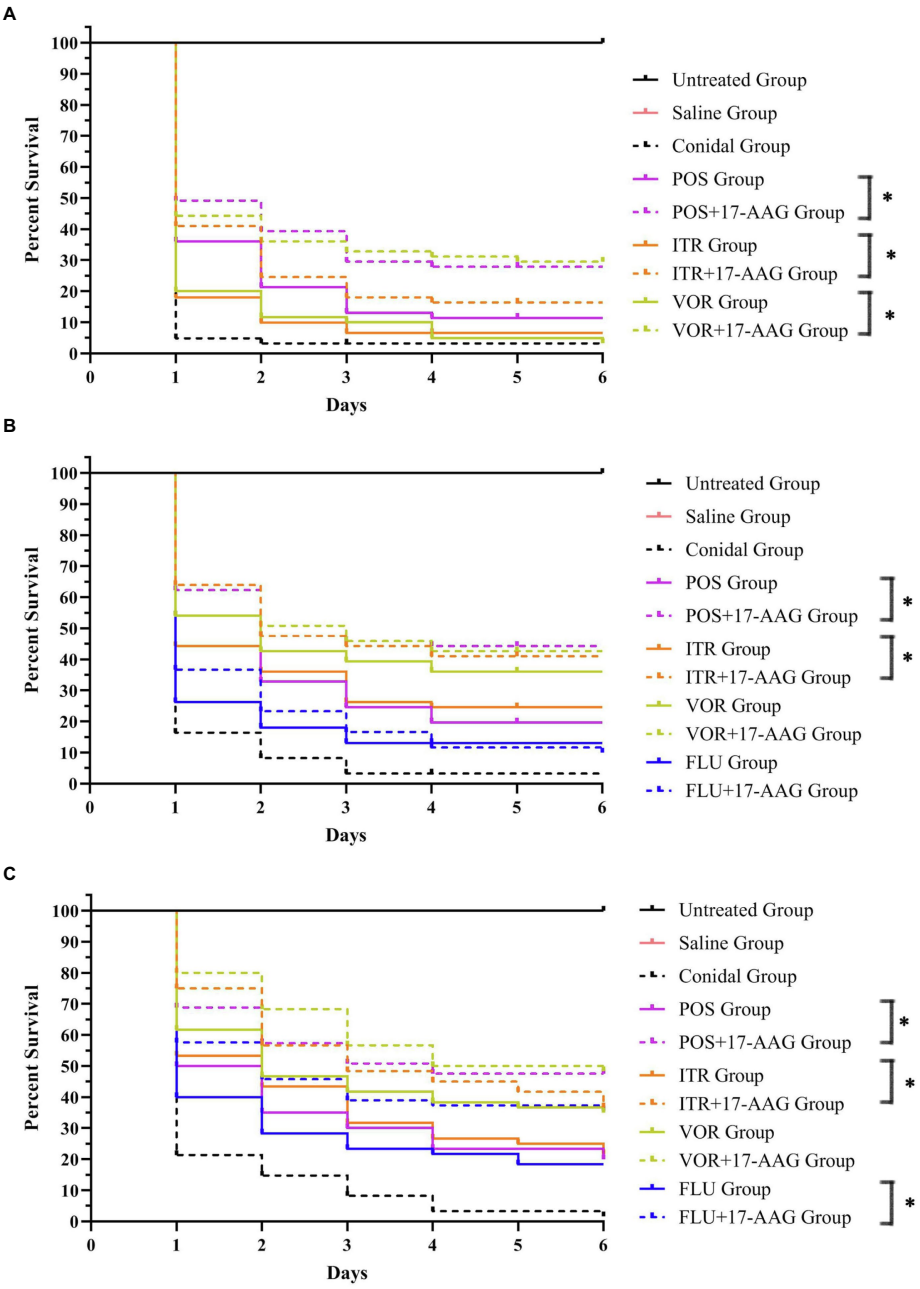
*Galleria mellonella* survival curves following infection with *Candida* spp. **(A)** *C. auris* AR 382; **(B)** *C. glabrate* 05448; and **(C)** *C. abicans* R2. Untreated group, wild-type uninfected larvae; Saline group, wild-type larvae injected with saline; Conidial group, larvae infected with *Candida* without any treatment; itraconazole (ITC) group, *Candida*-infected larvae treated with ITC alone; voriconazole (VOR) group, *Candida*-infected larvae treated with VOR alone; posaconazole (POS) group, *Candida*-infected larvae treated with POS alone; fluconazole (FLU) group, *Candida*-infected larvae treated with FLU alone; 17-AAG + ITC group, *Candida*-infected larvae treated with 17-AAG combined with ITC; 17-AAG + VOR group, *Candida*-infected larvae treated with 17-AAG combined with VOR; 17-AAG + POS group, *Candida*-infected larvae treated with 17-AAG combined with POS. 17-AAG + FLU group, *Candida*-infected larvae treated with 17-AAG combined with FLU; ^*^p < 0.05.

In this experiment, we used the caterpillar mellonella for the *in vivo* test, while the growth and survival rate of each group of larvae are shown in Figure 2. The survival rates of *C. abicans* R2, *C. auris* AR 382, and *C. glabrate* 05448 were significantly prolonged when 17-AAG combined with POS and 17-AAG combined with ITR. Especially when 17-AAG was used in combination with POS, the survival rate of *C. abicans* R2 was significantly improved (*p* < 0.05). When 17-AAG was used in combination with VOR, only *C. auris* AR 382 had significantly longer survival (*p* < 0.05). The 17-AAG + FLU combination can significantly prolong the survival time of larvae, especially *C. abicans* R2 (*p* < 0.05).

### Biofilm Drug Sensitivity Testing

**Figure 3:**
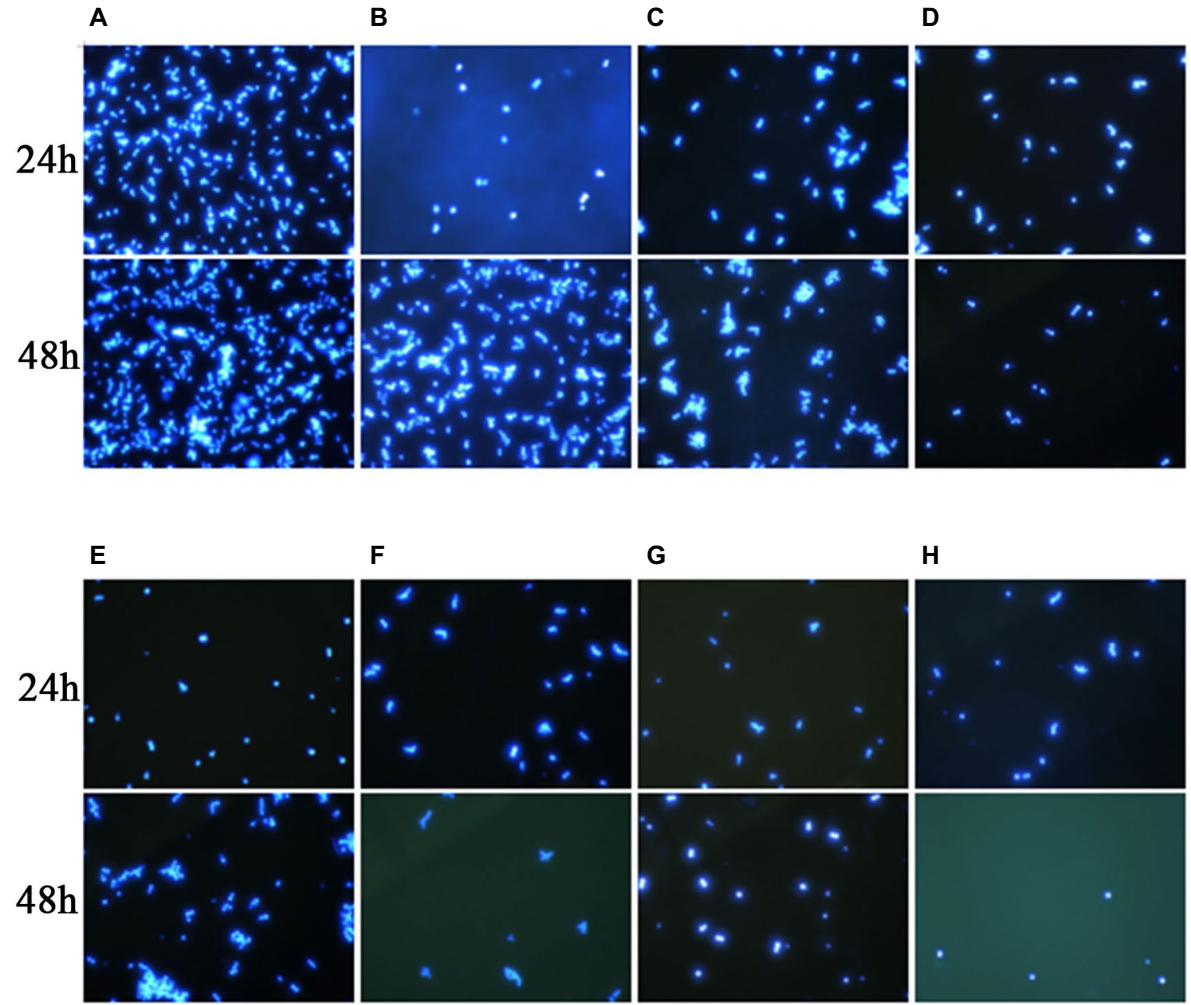
Effects of 17-AAG and azole monotherapy and in combination on biofilm formation by *C. auris* AR 382. **(A)** No drug; **(B)** 17-AAG; **(C)** ITR; **(D)** 17-AAG + ITR; **(E)** VOR; **(F)** 17-AAG + VOR; **(G)** POS; and **(H)** 17-AAG + POS.

In this experiment, we performed Calcofluor White staining of the test fungus, followed by observation under a fluorescence microscope. As shown in Figure 3, at 24 h, the *C. auris* AR 382 biofilm was significantly reduced in the monotherapy group compared with that in the drug-free group, while the biofilm reduction was more obvious in the combination group. At 48 h, the difference was more prominent, and the biofilms in the three drug combination groups were significantly reduced (Figure 3). After measuring the CFU value of different groups by paired *T* test, we achieved consistent results (Table 3). We noted that 17-AAG could play an antifungal role by inhibiting the production of fungal biofilms when used in combination with azole drugs.

**Table 3:** Concentration of spores in *C. auris* AR 382 biofilms.

Group	Spore concentration (CFU/ml)	
	24 h	48 h
No drug	3.40 × 10^6^	5.98 × 10^6^
17-AAG	3.22 × 10^6^	9.35 × 10^6^
ITR	1.48 × 10^6^	5.53 × 10^5^^*^
17-AAG + ITR	1.30 × 10^6^	6.57 × 10^5^^*^
VOR	7.43 × 10^5^	1.11 × 10^6^^*^
17-AAG + VOR	7.10 × 10^5^^*^	8.20 × 10^5^^*^
POS	1.15 × 10^6^^*^	6.53 × 10^5^^*^
17-AAG + POS	3.67 × 10^5^^*^	2.80 × 10^5^^*^

* The CFU values of the group without antifungal drugs and the antifungal drugs group were statistically significant (*p* < 0.05).

### Extracellular R6G Testing

**Figure 4:**
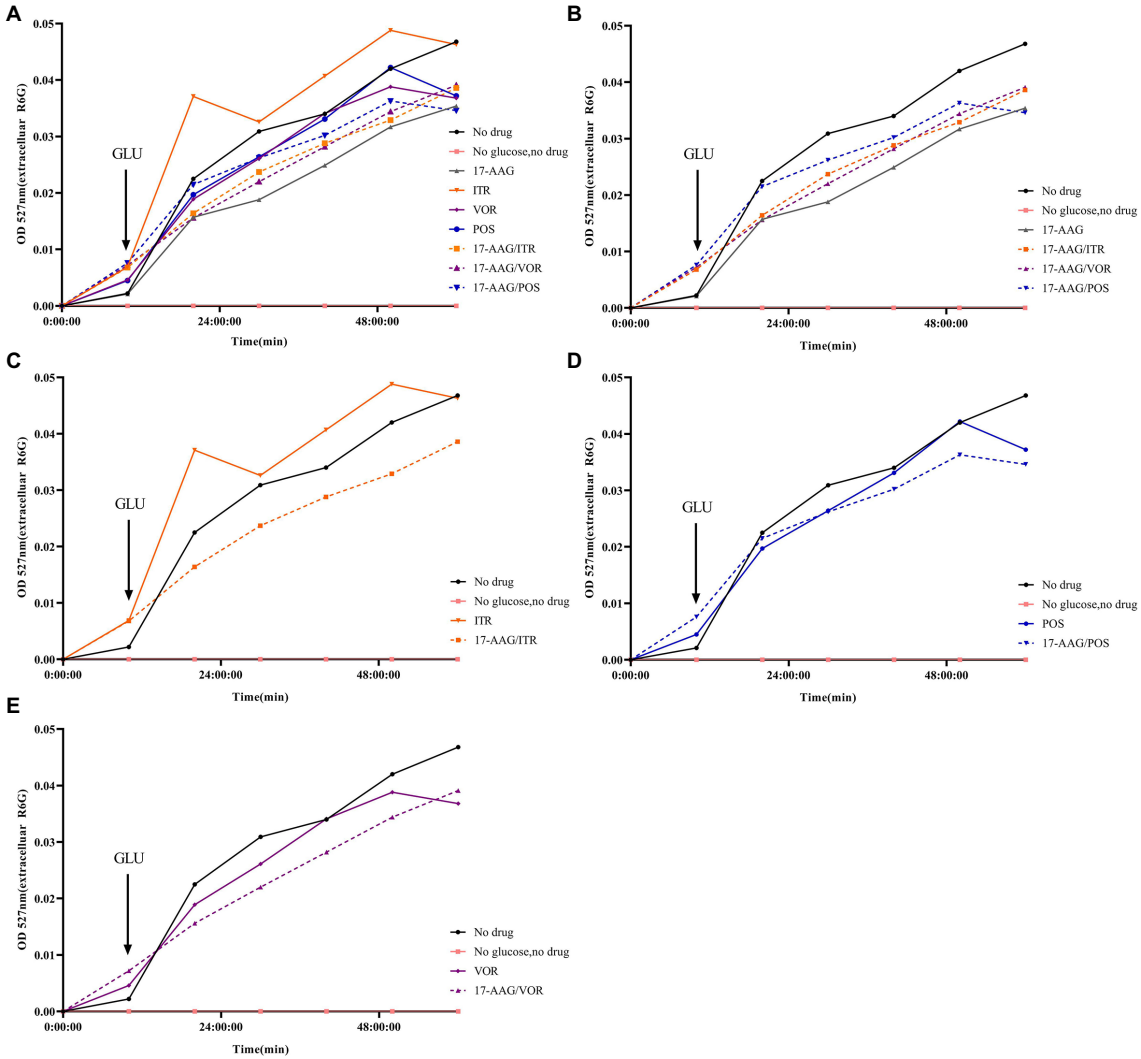
Extracellular R6G measurements of 17-AAG and azole alone or in combination. **(A)** No drug+No glucose+17-AAG + ITR + VOR + POS + 17-AAG/ITR + 17-AAG/VOR + 17-AAG/POS; **(B)** No drug+No glucose+17-AAG + 17-AAG/ITR + 17-AAG/VOR + 17-AAG/POS; **(C)** No drug+No glucose+ITR + 17-AAG/ITR; **(D)** No drug+No glucose+VOR + 17-AAG/VOR; and **(E)** No drug+No glucose+POS + 17-AAG/POS.

In our experiment, the fluorescent dye R6G was used, which is known to be transported in and out of several multi-drug resistant yeast cells to mammalian cells. Therefore, for the detection purpose, a spectrophotometer was used to analyze R6G for *C. auris* AR 382 under different drug treatments, followed by comparative analyses. When the cells in all groups were cultured in glucose-free PBS, the content of extracellular R6G did not increase. After the addition of glucose at 10 min, the extracellular OD value of each group began to increase to varying degrees. When all groups were compared with the sugar-free and drug-free groups, the following results were obtained: 1: in the sugar-free group, no intracellular energy was present to drive the efflux pump, which resulted in extremely low extracellular R6G content. 2: In the drug-free treatment group, significant efflux of R6G occurred when glucose was added to drive the efflux pump. The effluence of R6G in 17-AAG and azoles alone groups was less than that in the control group. However, the combination of 17-AAG with azoles drugs resulted in obviously decreased efflux of R6G (Figure 4). Thus, our results confirmed that 17-AAG when used in combination with an antifungal drug, possibly by inhibiting the efflux pump, decreasing the antifungal drug efflux, drug accumulation in the cells, as well as the subsequent reaction to fungal cell death.

### Intracellular ROS Testing

**Figure 5:**
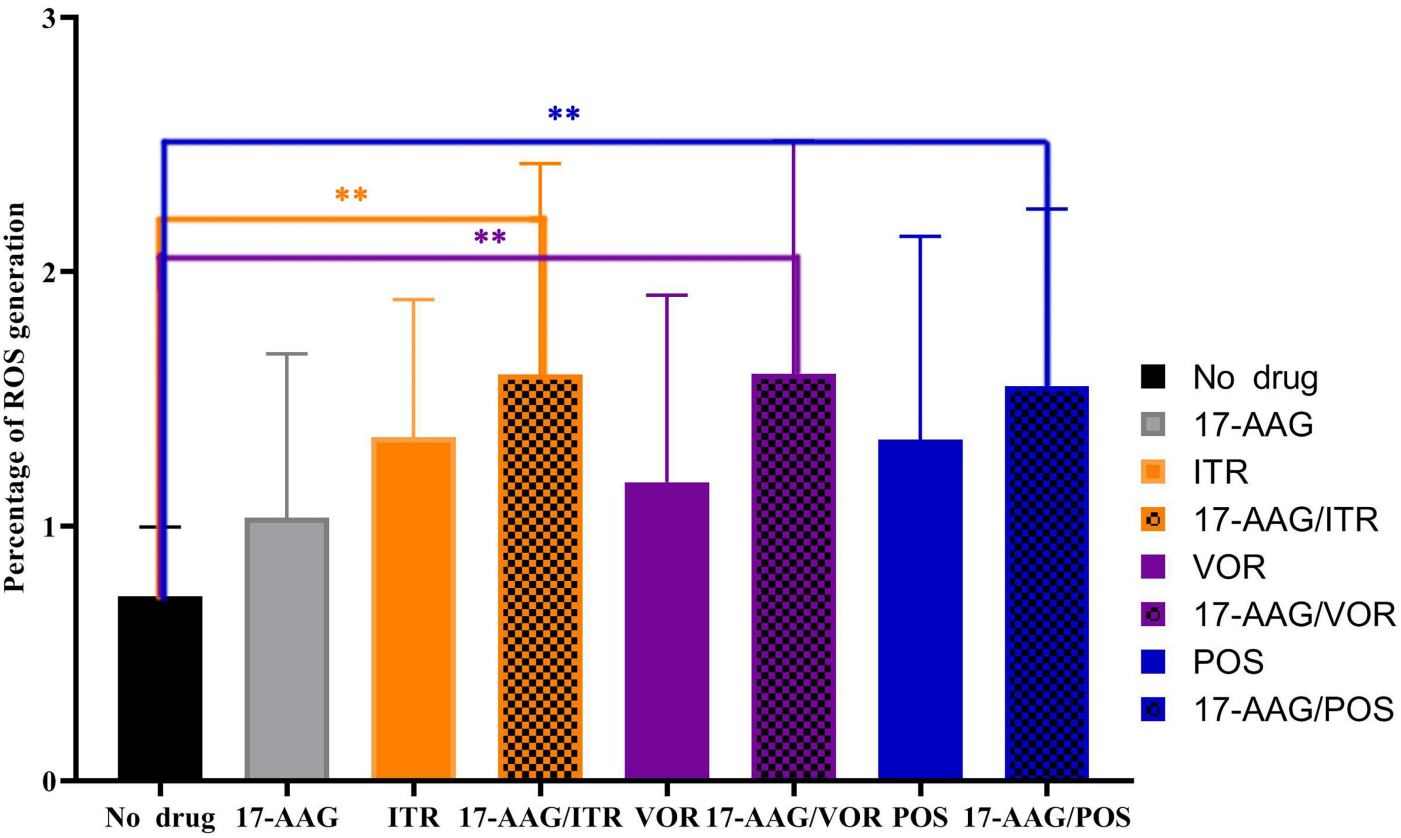
17-AAG and azole alone and in combination, extracellular ROS production level. ^**^p < 0.0.

In this study, we used the stain DHR-123—an uncharged and fluorescent-free intracellular ROS detection agent—that could actively penetrate the cell membranes. It can be oxidized to rodamine-123 by intracellular ROS that emit bright green fluorescence, which can be quantitatively detected by flow cytometry. Three repeated measurements revealed that the ROS production in *C. auris* AR 382 was higher when monotherapy was performed relative to that in the drug-free control group. However, when 17-AAG was used in combination with azole, the proportion of ROS-producing cells was significantly increased when compared with that of the control cells (*p* < 0.05; Figure 5). These results suggest that, when drugs accumulate in fungal cells, the subsequent accumulation of ROS induces the apoptosis of fungal cells.

## Discussion

Some past researches have demonstrated that 17-AAG combined with azoles or echinosporins exhibit effective synergistic antifungal activity against *Aspergillus fumigatus, Aspergillus terreus, Candida rubra,* and *Candida albicans* under both *in vitro* and *in vivo* conditions (Blum et al., 2013; Jacob et al., 2015; Chatterjee and Tatu, 2017; Refos et al., 2017). In addition, some previous studies have reported that 17-AAG can significantly reduce the ATPase activity in the *in vitro* cryptococcus infection model at 37°C, which changes the effectiveness of azole drugs range from ineffective to highly effective. However, information about the effect of *C. auris* and some azole-resistant *Candida* is limited in the literature. Therefore, the application of 17-AAG combined with anti-fungal agents in the treatment of fungal infection has significant potential.

Although 17-AAG alone demonstrated non-activity against some azole-resistant *Candida* spp., for azole-resistant *Candida* spp., it demonstrated a synergistic effect with ITR, VOR, and POS, especially with POS in combination with azoles under *in vitro* conditions. For FLU-resistant *Candida* sp., it demonstrated a synergistic effect with FLU. The effective working range of 17-AAG was found to be 0.5–32 μg/ml. Our results provide strong evidence for the potential value of 17-AAG in the treatment of fungal infections caused by *C. auris* and some azole-resistance *Candida*.

We used the caterpillar mellonella as a model in our *in vivo* experiments because these larvae demonstrate similar reactions to those of mammals, making them ideal for use in our *in vivo* experiments (Tan et al., 2021). Our *in vivo* test results were nearly the same as those of our *in vitro* test results. In other words, 17-AAG demonstrated better fungicidal effect when combined with azole drugs. However, in order to validate our *in vitro* test results, further experiments with mice need to be conducted.

Some past studies have proved that 17-AAG can reduce the ATPase activity by competitively binding to the ADP/ATP site with Hsp90. For instance, the IC 50 of 37°C was lower than that of 25°C in a model of *Cryptococcus* infection *in vitro* (Chatterjee and Tatu, 2017). Lv has demonstrated that 17-AAG could competitively bind to the ADP/ATP site of Hsp90 in order to prevent the formation of the Hsp90 chaperone complex, which then leads to the development of unfolded substrate and further degradation (Lv et al., 2020). Further study revealed that the synergistic action target of 17-AAG and azole in anti-azole drug-resistant fungi may inhibit the drug efflux pump on fungal cell membranes, possibly caused by the inhibition of the activity of ATPase inherent in fungal cells by 17-AAG. These drugs accumulate in fungal cells. As 17-AAG stimulates the production of Ca^2+^ and endoplasmic reticulum kinase-dependent ROS in the human body (Mitchell et al., 2007), the combination of 17-AAG and azole in this study induced ROS accumulation in the fungal cells, which is consistent with the results of human experiments. Aon confirmed that ROS may exert harmful effects in either a low or a high proportion of cells. When excessive ROS accumulates in cells, the cells cannot be regulated (Aon et al., 2012). ROS has been confirmed to play the main role in the regulation of the cell cycle progression (Verbon et al., 2012), and the cells mediated by ROS over a long time are known to undergo pathological death (Zorov et al., 2014). In addition, ROS can further induce the accumulation of intracellular hydroxyl radicals, which is the key factor of cell apoptosis (Haruna et al., 2002). Therefore, when 17-AAG was used in combination with azole, the fungal cell apoptosis was negatively correlated with the drug efflux and positively correlated with the accumulation of intracellular ROS, which in turn reduced the resistance of fungi. Our study provides meaningful clues toward the elucidation of the mechanism of 17-AAG-specific antifungal activities.

When compared with geldanamycin, 17-AAG exhibited a higher stability, better bioavailability, and greater water solubility (Li et al., 2020b), which allows its wider distribution across animal tissues, thereby signifying its greater potential in clinical applications. Recent studies have demonstrated that low concentration of 17-AAG can inhibit the growth of breast cancer cells, induce apoptosis (Esgandari et al., 2021), and prevent myocardial dysfunction after myocardial infarction in rats (Marunouchi et al., 2021). HSP90 is a host-dependent factor of human coronaviruses MERS-CoV SARS-COV and SARS-COV-2, while HSP90 inhibitors can be used as an effective broad-spectrum antiviral agent against human coronaviruses (Li et al., 2020a). In addition, a Chinese group verified, for the first time, that the synthesis of a new 17-AAG glucoside by UDP glycosyltransferase *in vitro* can improve its water solubility and anti-tumor efficacy, thereby proving that it can be applied to clinical development through the prodrug method (Woo et al., 2017; Li et al., 2020b). Although there are more optimistic improvement programs available, the number of supporting clinical and experimental data is scarce. Presently, we need to invest more efforts toward the prevention and treatment of this type of fungal infection.

## Conclusion

17-AAG offers great potential to reduce azoles resistance in fungi under *in vitro* conditions, which suggests that the use of Hsp90 inhibitors in combination with the existing agents for *Candida* infection is a potential therapy. Nevertheless, further investigations are warranted to clarify its potential clinical application.

## Data Availability Statement

The original contributions presented in the study are included in the article/Supplementary Material, further inquiries can be directed to the corresponding authors.

## Author Contributions

XZ carried out the *in vitro* antifungal experiment. LuL did other experiments to explore the mechanism. LuL and JT conducted *in vivo* antifungal experiments. SK and LT collection and analysis the experiment data. YS and JM designed, interpreted the experiment data, and wrote the manuscript. YS, LiL, and HZ revised the manuscript critically for important content. All authors contributed to the article and approved the submitted version.

## Funding

This work was supported by Grant no. WJ2021M261 (YS) from Health Commission of Hubei Province Scientific Research Project and Grant no. 2019CFB567 (YS) from the Natural Science Foundation of Hubei Province. The funders had no role in study design, data analysis, the decision to publish, or preparation of the manuscript.

## Conflict of Interest

The authors declare that the research was conducted in the absence of any commercial or financial relationships that could be construed as a potential conflict of interest.

## Publisher’s Note

All claims expressed in this article are solely those of the authors and do not necessarily represent those of their affiliated organizations, or those of the publisher, the editors and the reviewers. Any product that may be evaluated in this article, or claim that may be made by its manufacturer, is not guaranteed or endorsed by the publisher.
