# Supplementary figures and images for: A Preliminary in vitro and in vivo Evaluation of the Effect and Action Mechanism of 17-AAG Combined With Azoles Against Azole-Resistant Candida spp
Source: Front Microbiol. 2022 Jul 7;13:825745. doi: 10.3389/fmicb.2022.825745 (PMC9300965; doi:10.3389/fmicb.2022.825745)

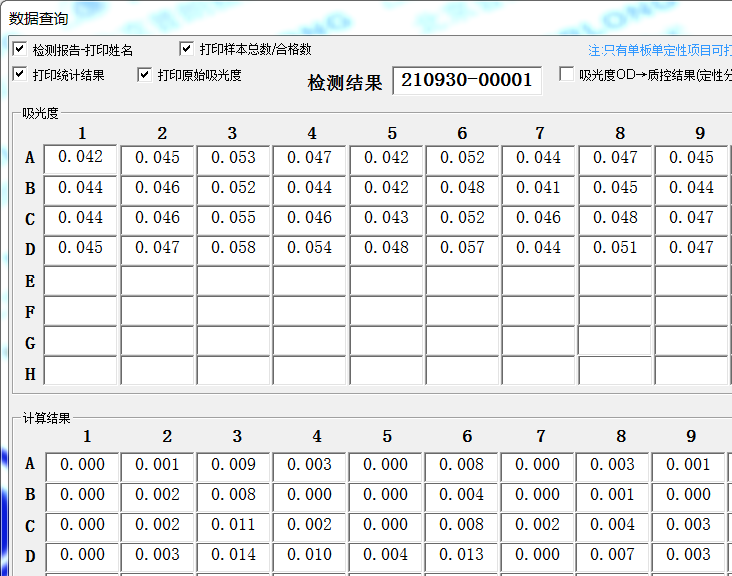

Supplement: Supplementary file 1 [file Data_Sheet_1.ZIP › Supplementary Material Presentation0615/R6G实验原始数据/9.30/0.png]

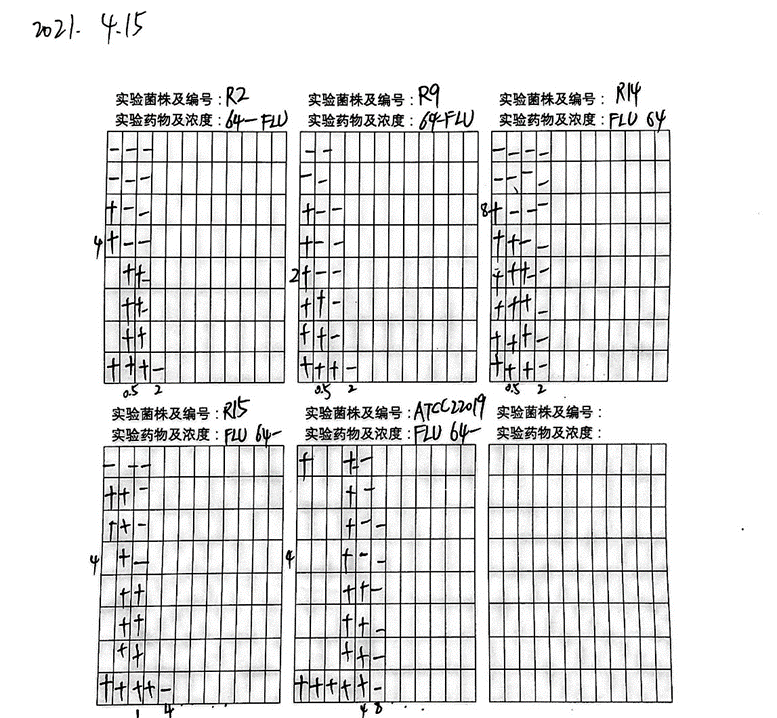

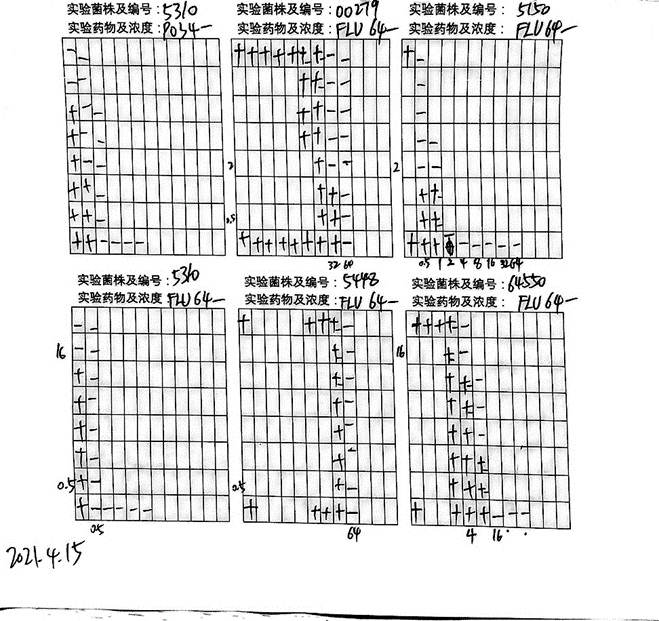

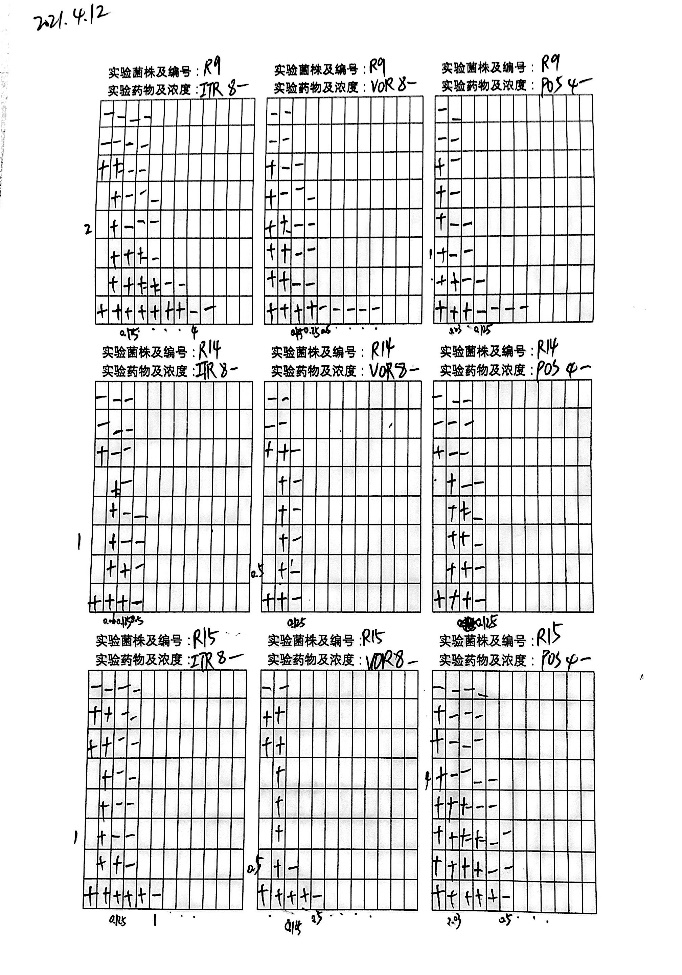

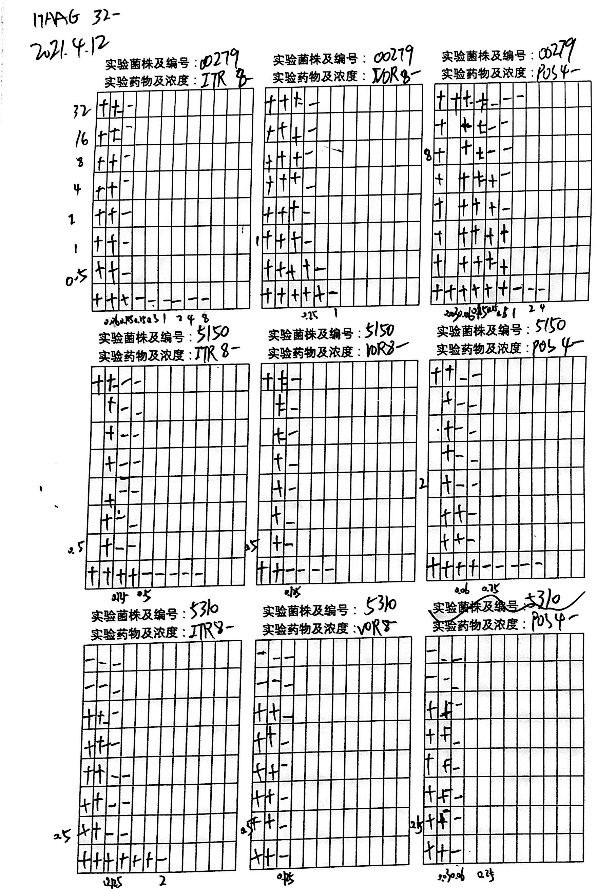

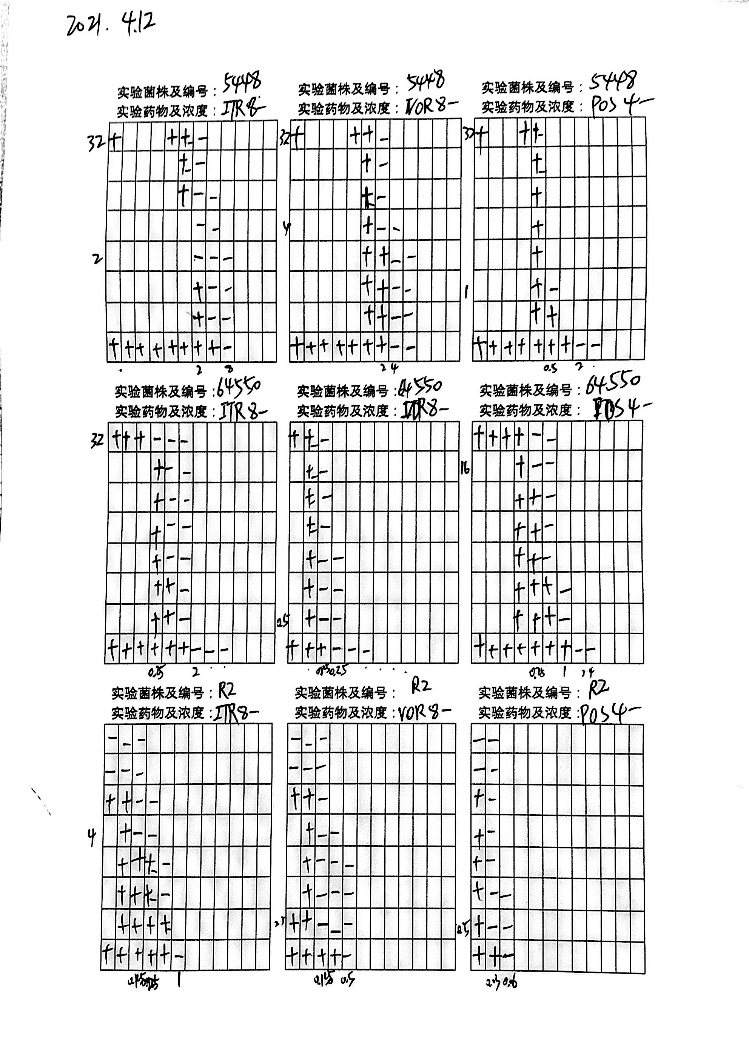

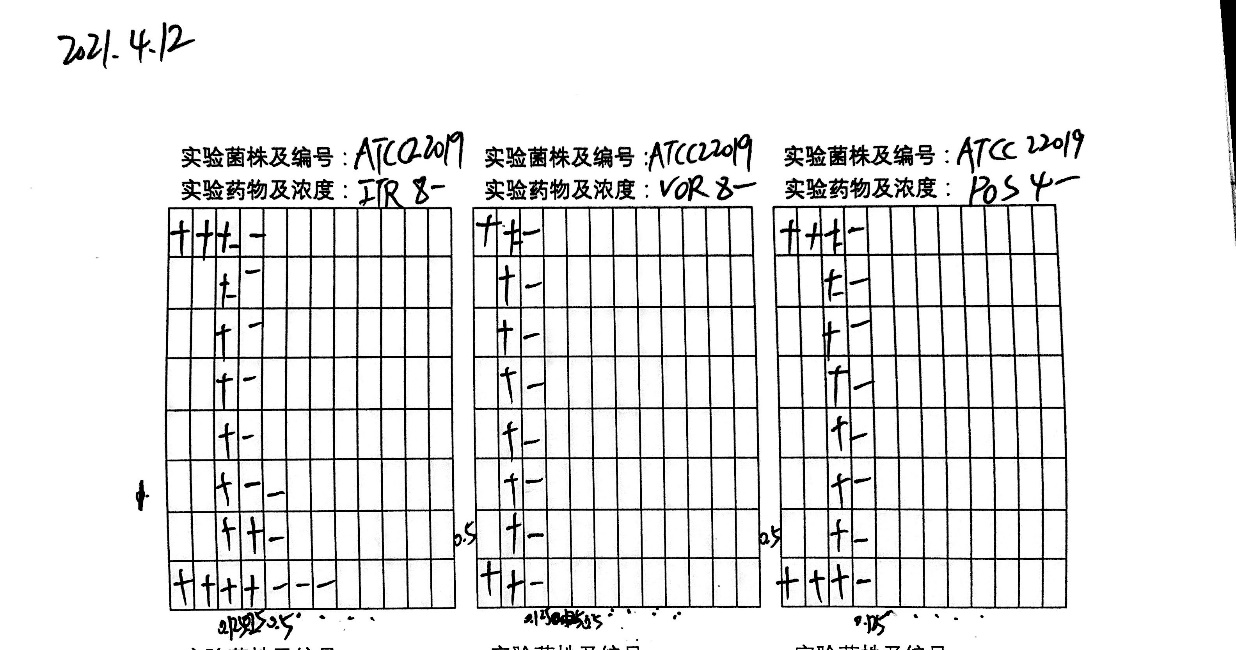

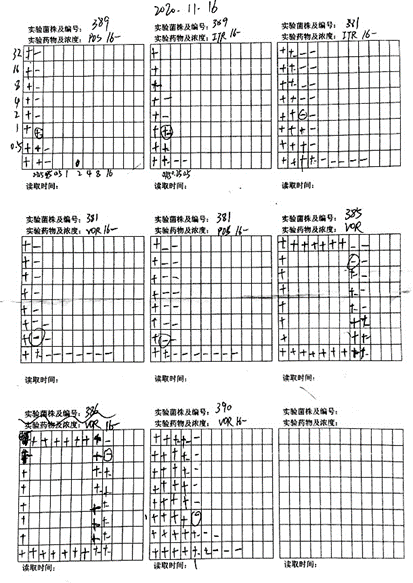

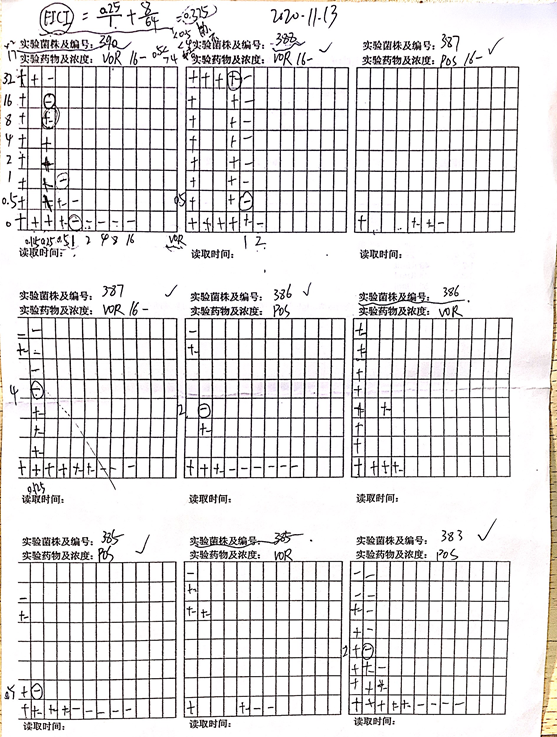

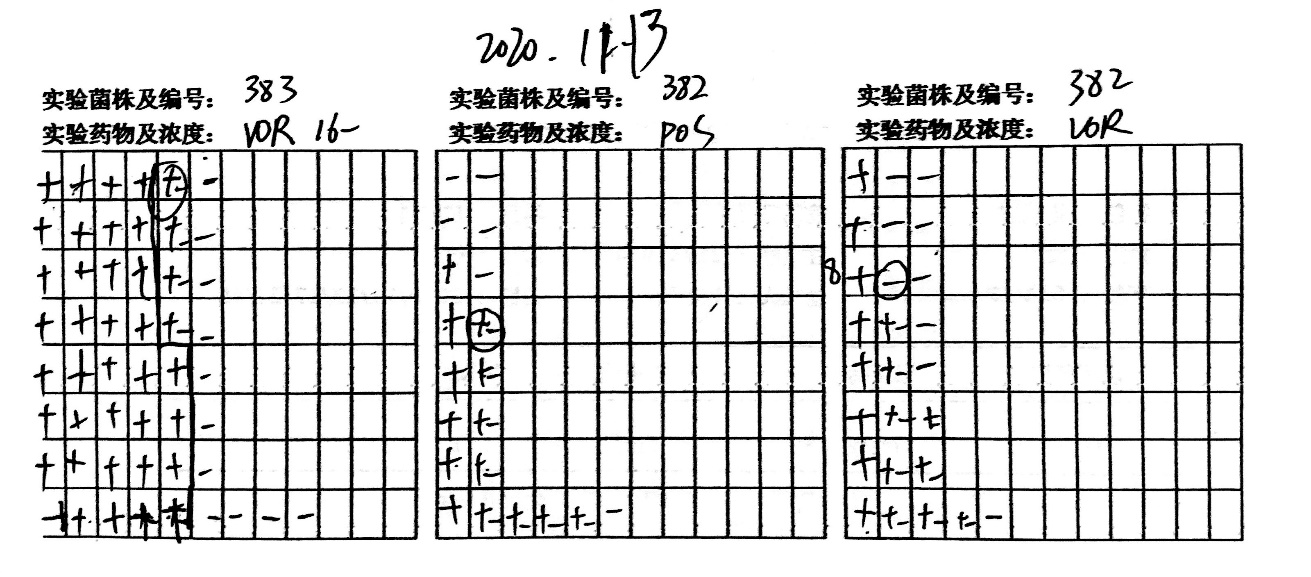


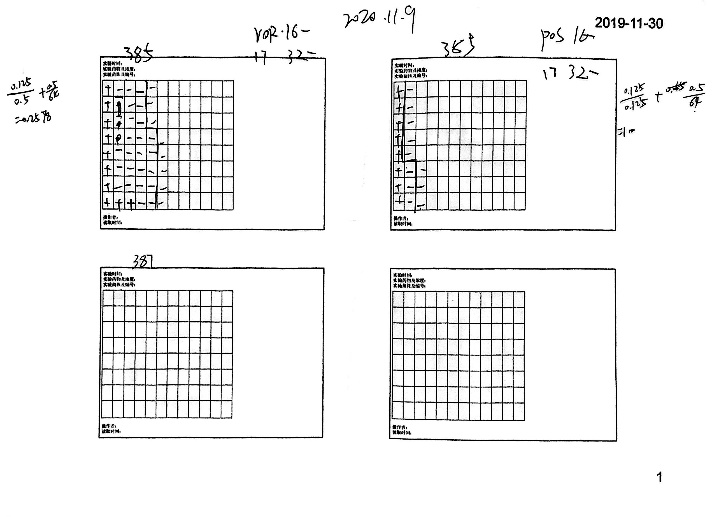

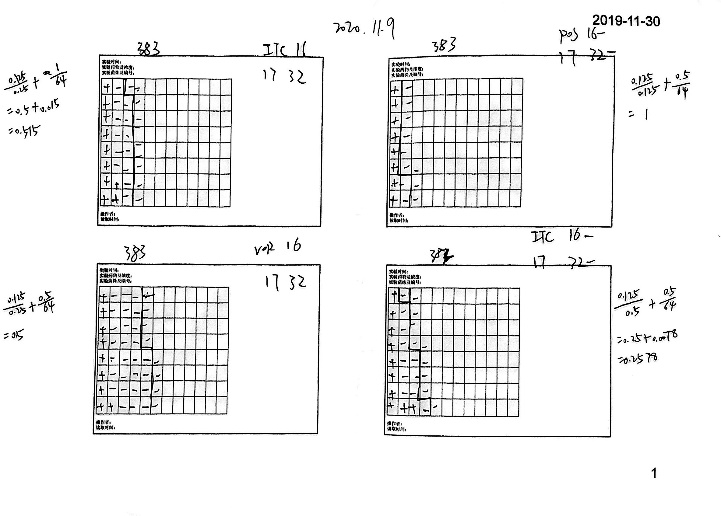

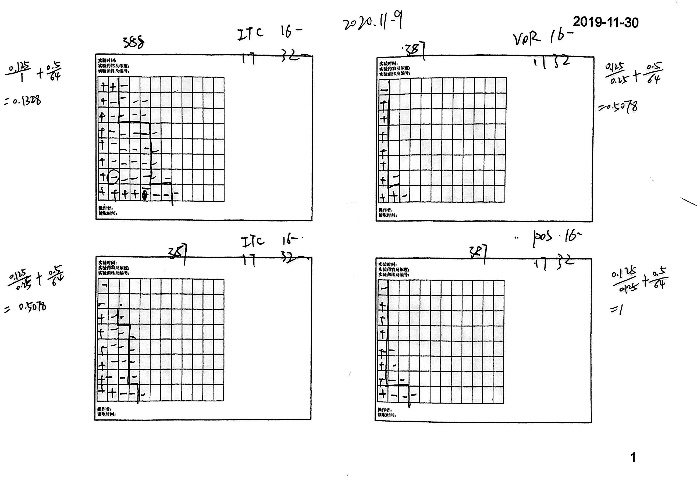

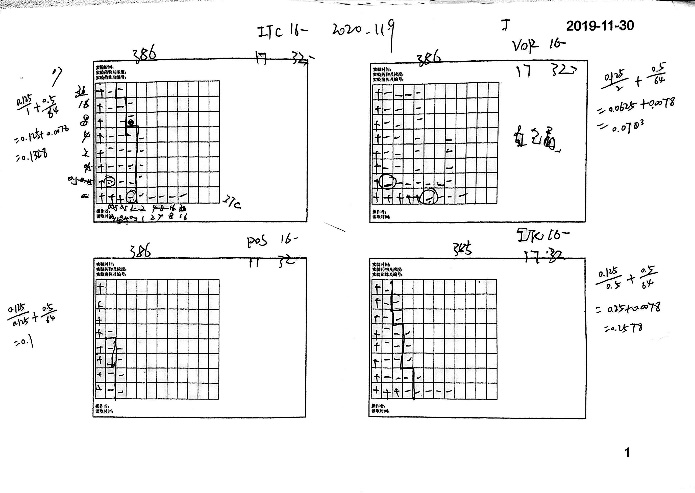

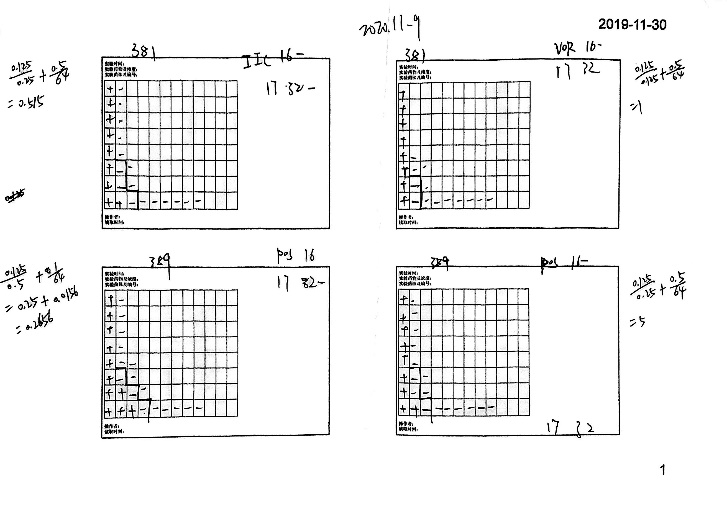

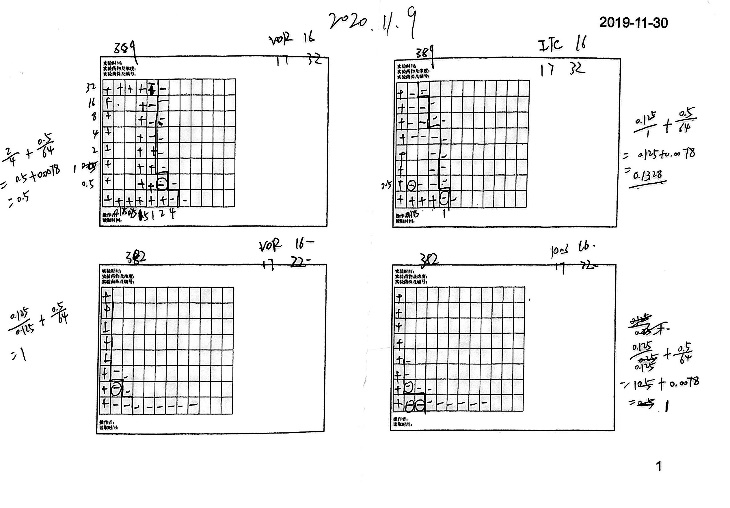

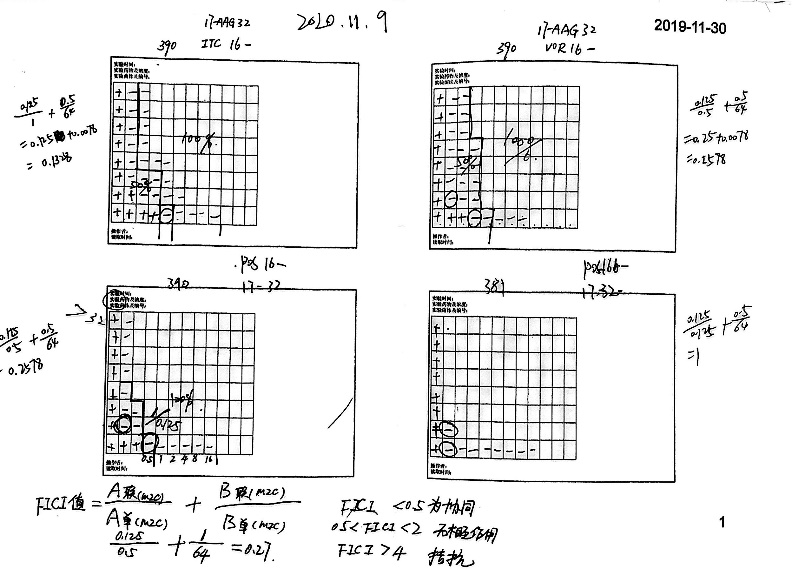

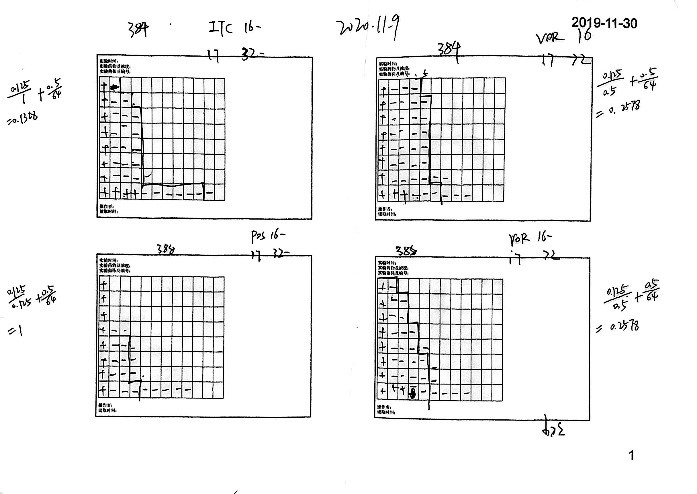

Supplement: Supplementary file 1 [file Data_Sheet_1.ZIP › Supplementary Material Presentation0615/体外实验原始数据/体外实验原始数据.docx]

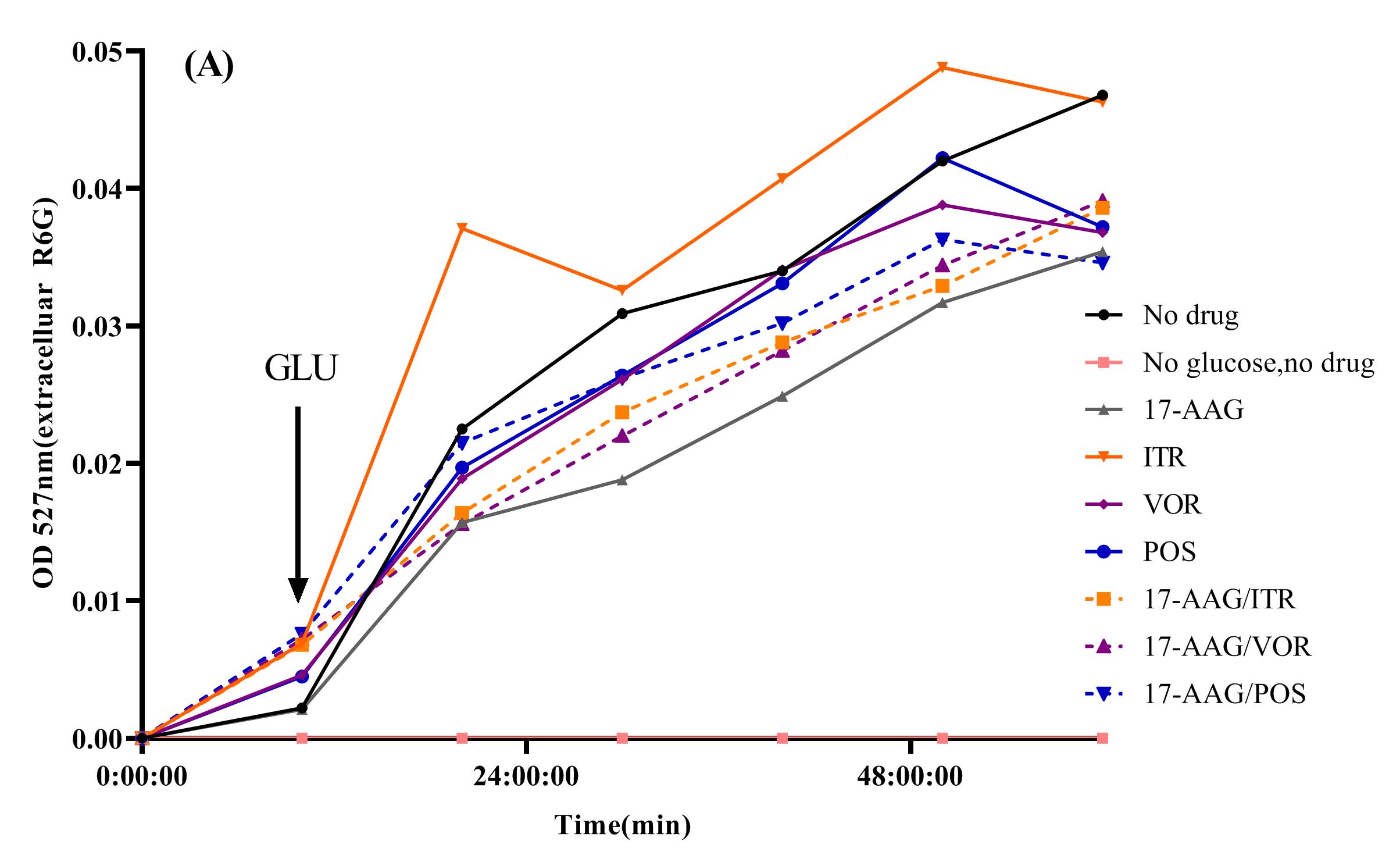

Supplement: Supplementary file 1 [file Data_Sheet_1.ZIP › Supplementary Material Presentation0615/新图片/11.jpg]

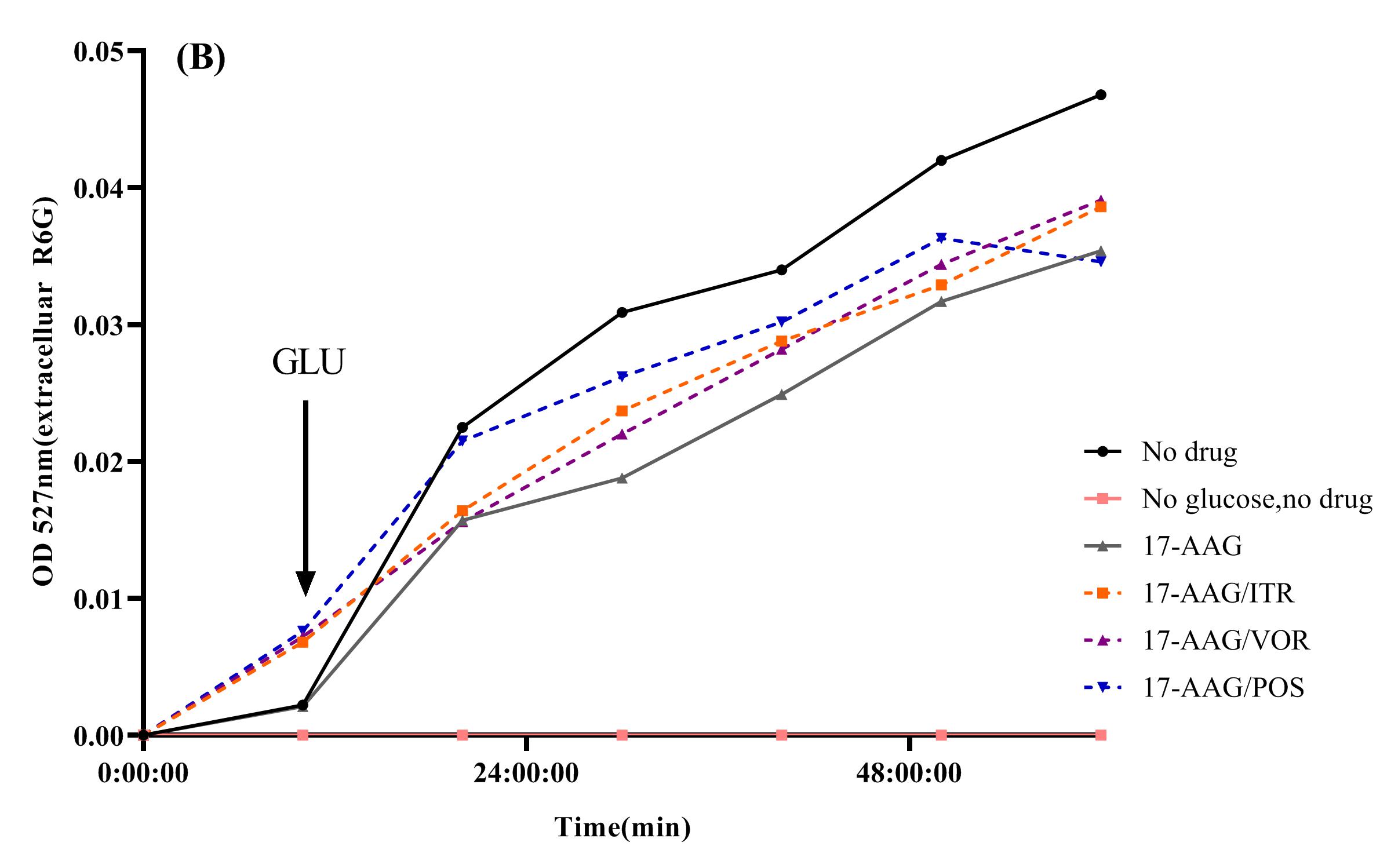

Supplement: Supplementary file 1 [file Data_Sheet_1.ZIP › Supplementary Material Presentation0615/新图片/12.jpg]

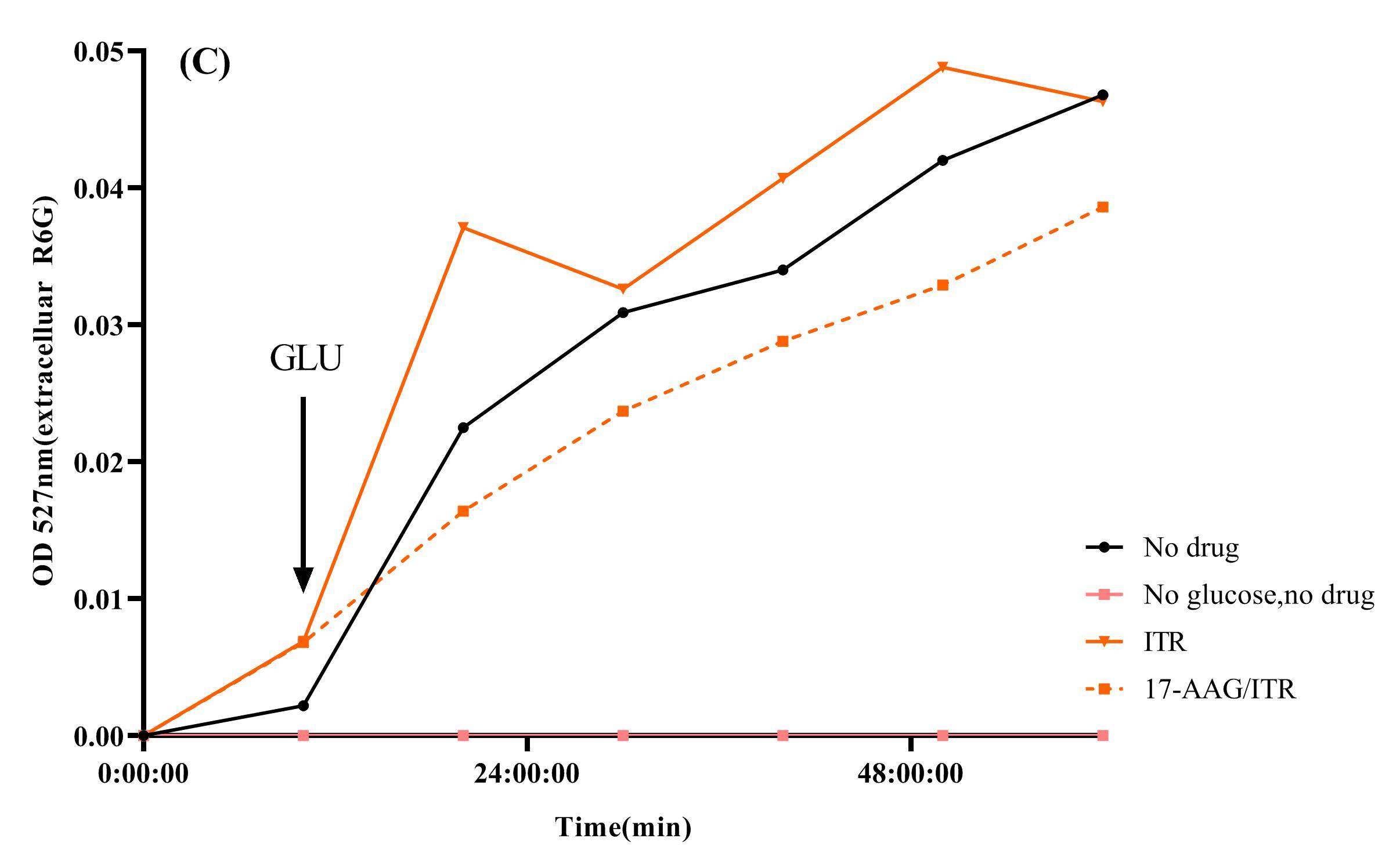

Supplement: Supplementary file 1 [file Data_Sheet_1.ZIP › Supplementary Material Presentation0615/新图片/13.jpg]

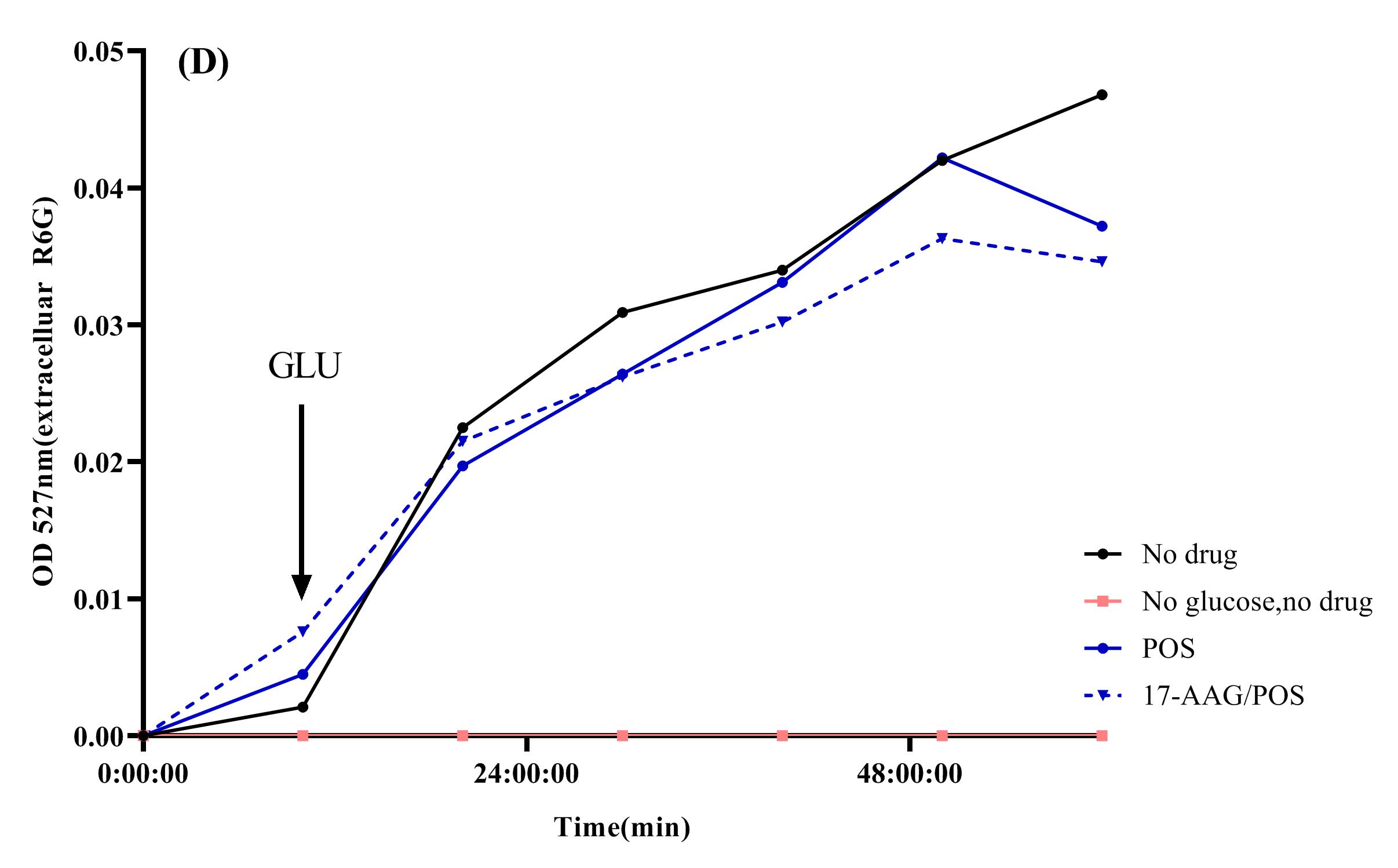

Supplement: Supplementary file 1 [file Data_Sheet_1.ZIP › Supplementary Material Presentation0615/新图片/14.jpg]

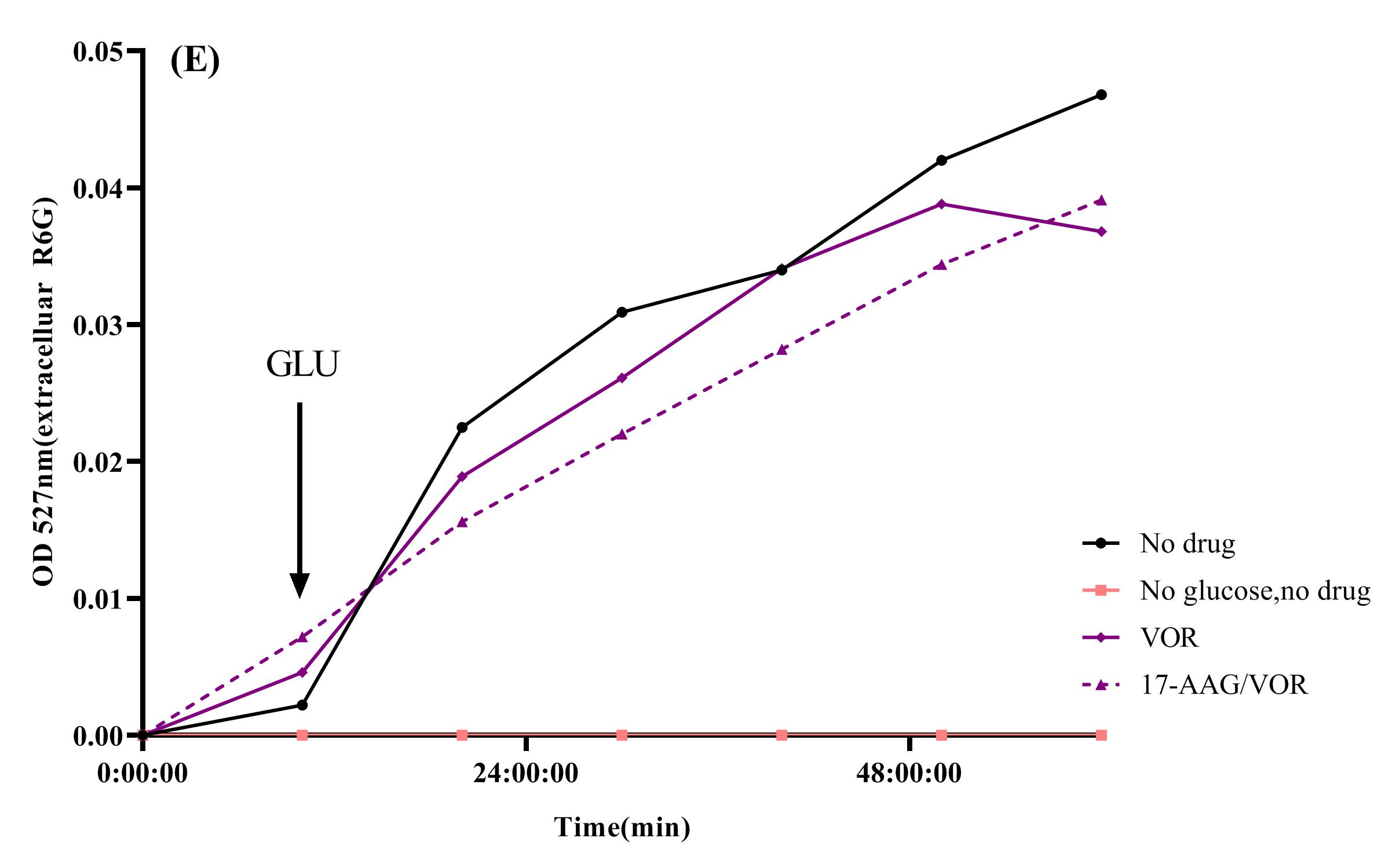

Supplement: Supplementary file 1 [file Data_Sheet_1.ZIP › Supplementary Material Presentation0615/新图片/15.jpg]

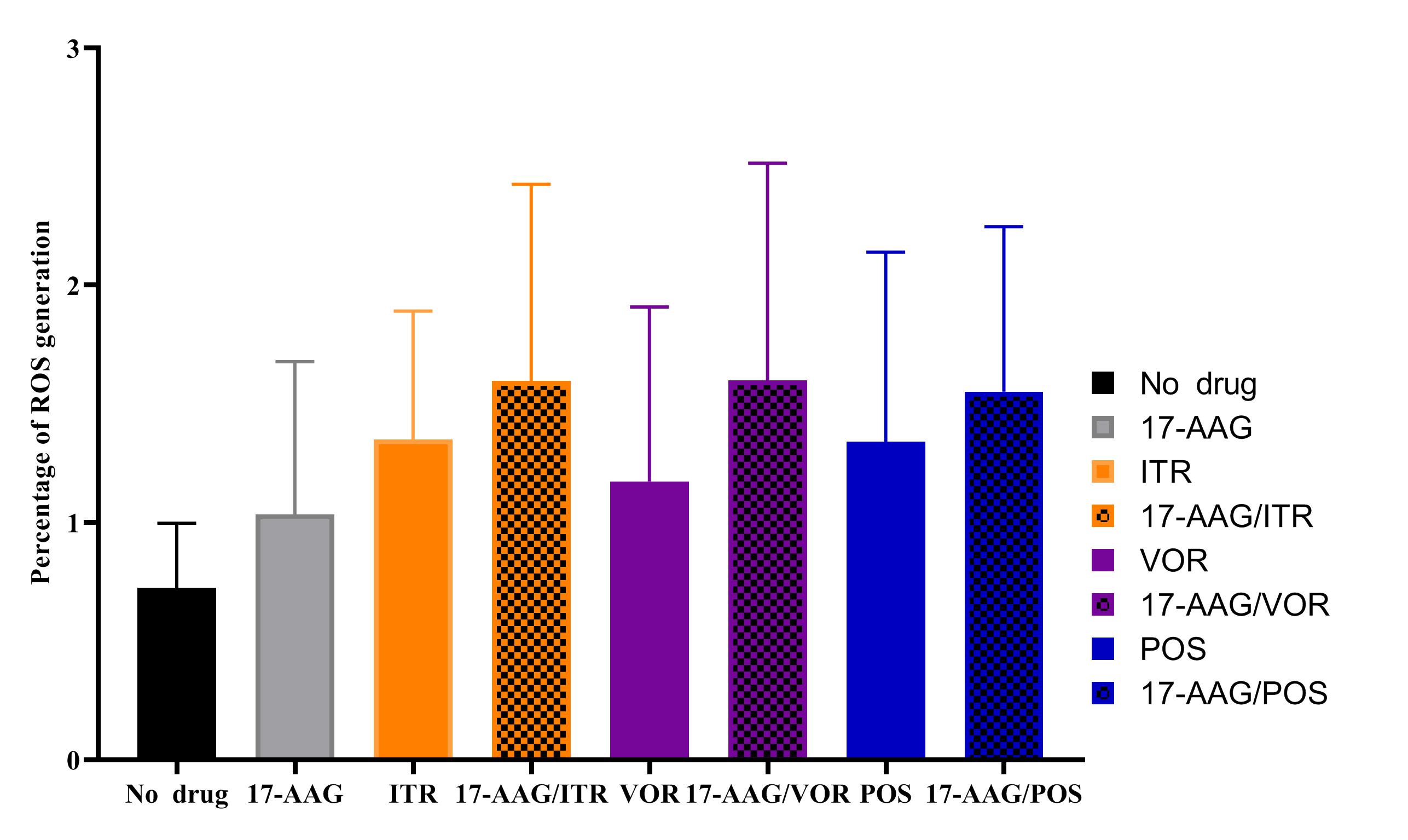

Supplement: Supplementary file 1 [file Data_Sheet_1.ZIP › Supplementary Material Presentation0615/新图片/16.jpg]

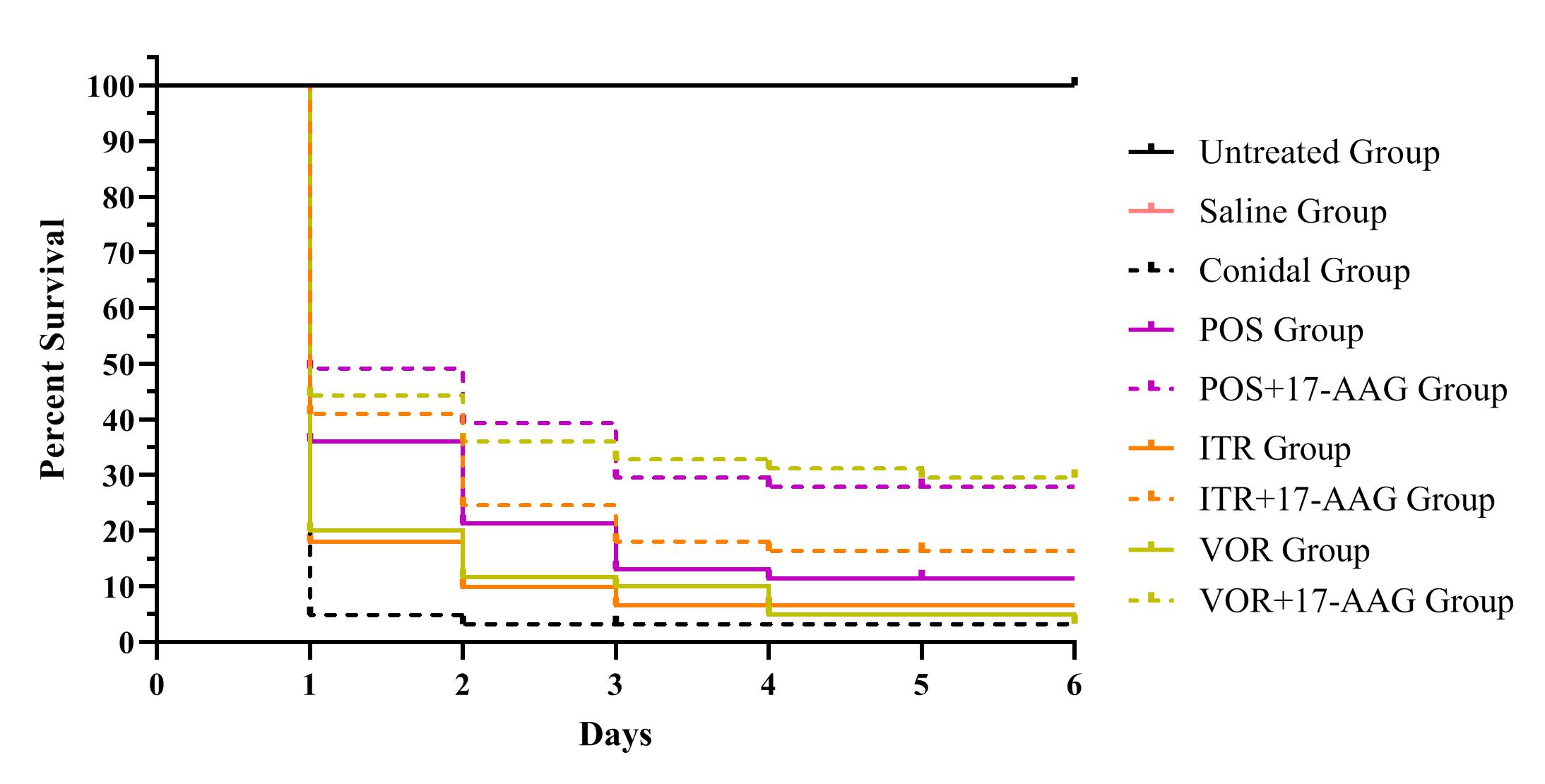

Supplement: Supplementary file 1 [file Data_Sheet_1.ZIP › Supplementary Material Presentation0615/新图片/382 a.jpg]

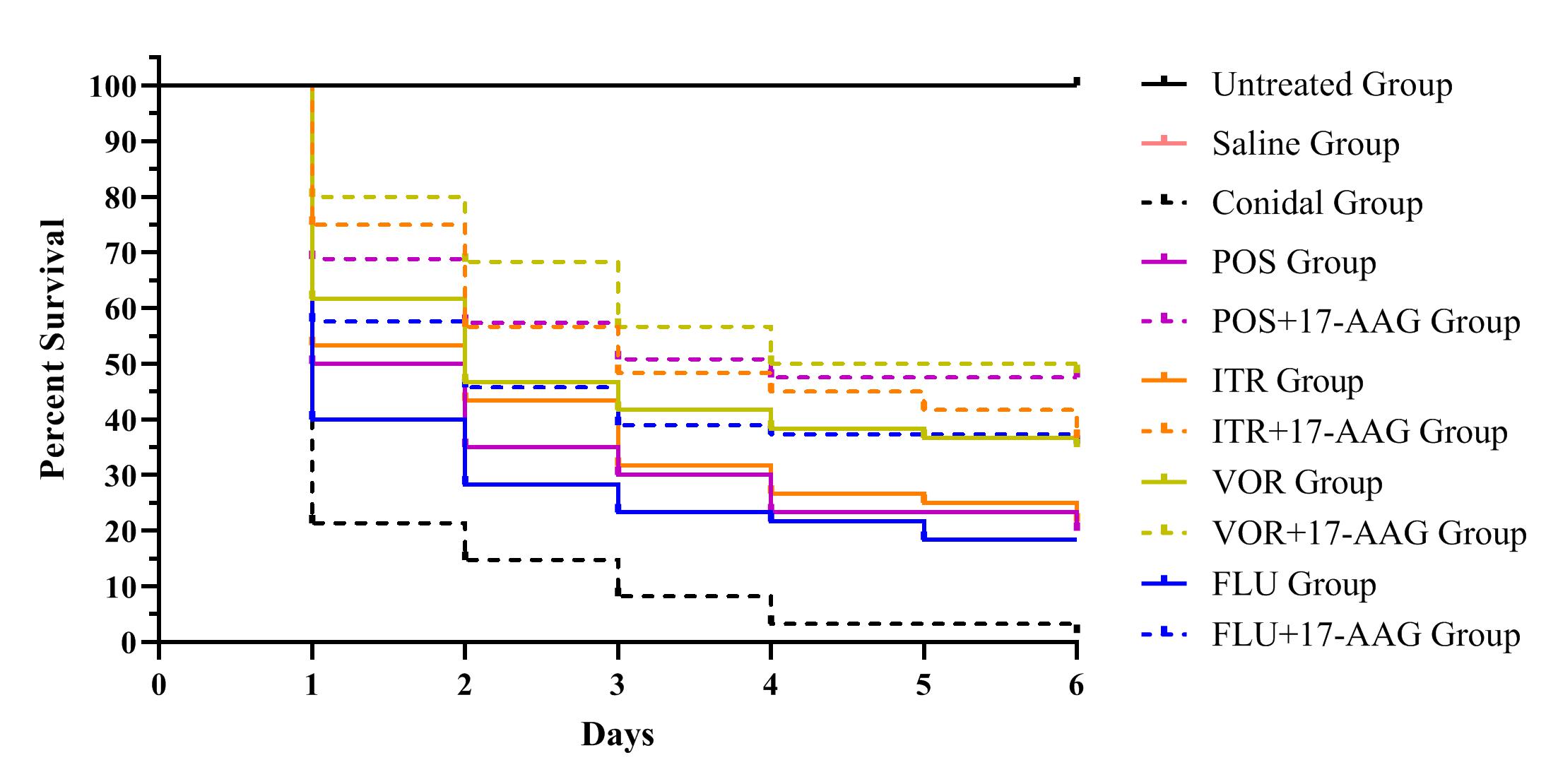

Supplement: Supplementary file 1 [file Data_Sheet_1.ZIP › Supplementary Material Presentation0615/新图片/abicans C.jpg]

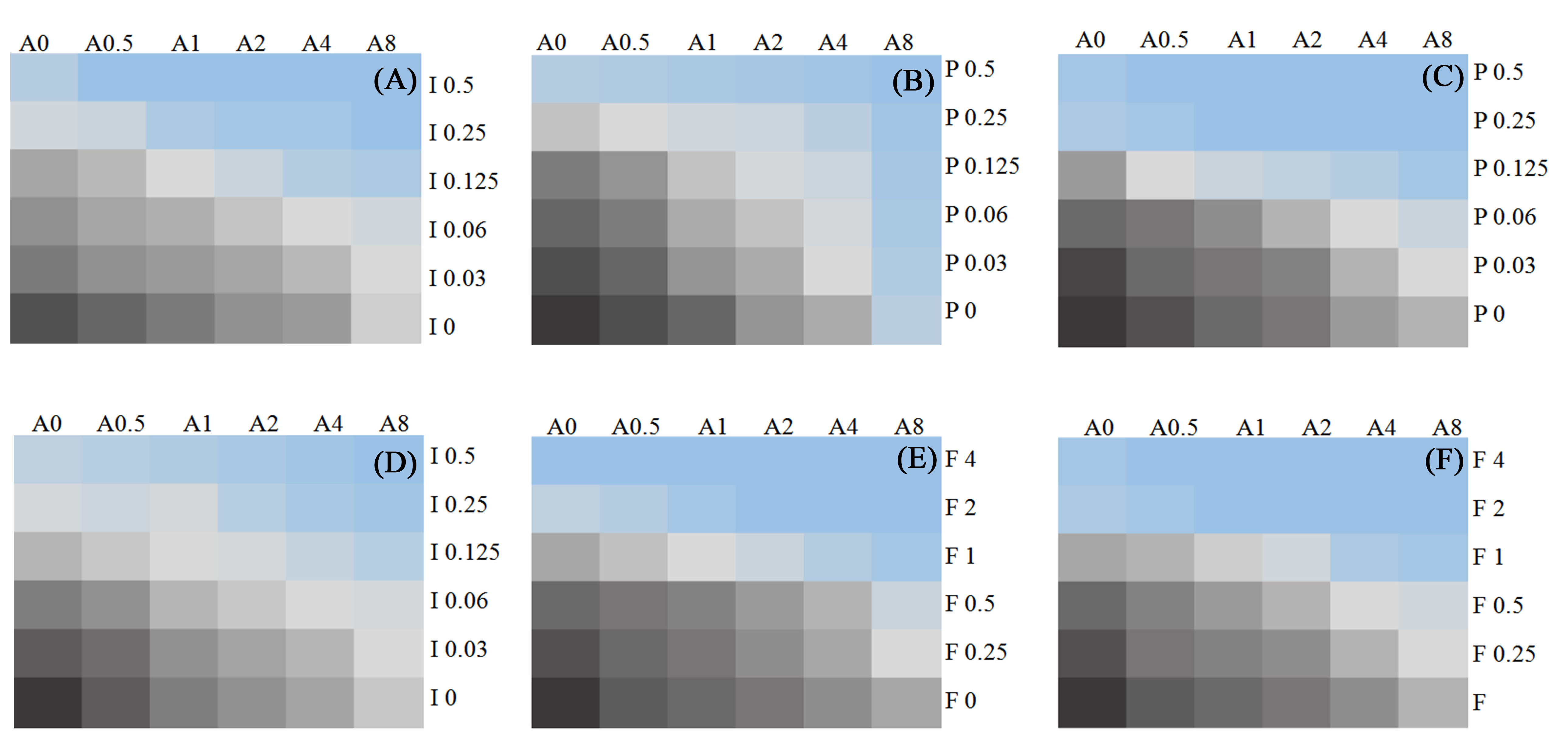

Supplement: Supplementary file 1 [file Data_Sheet_1.ZIP › Supplementary Material Presentation0615/新图片/figure 1.jpg]

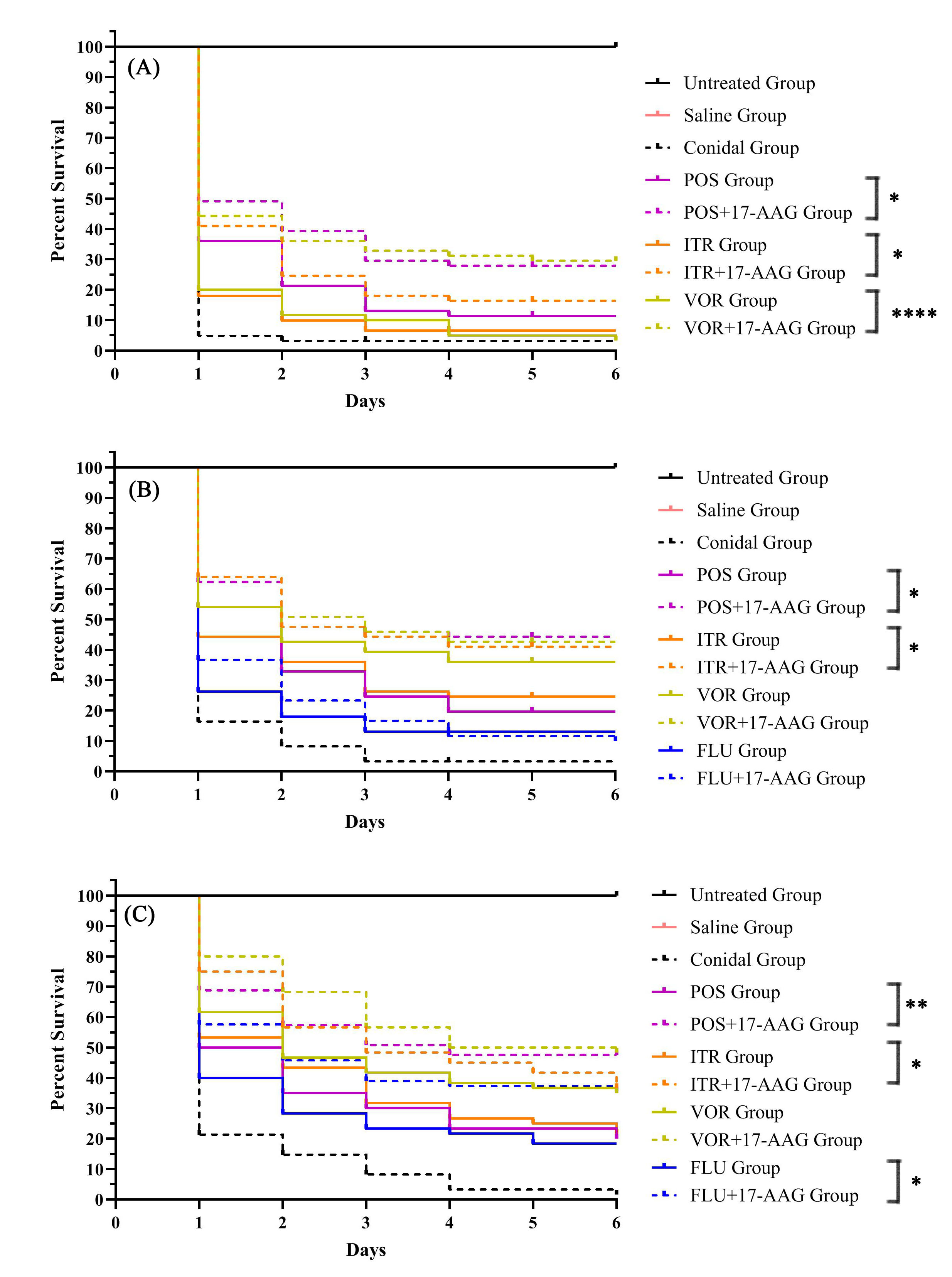

Supplement: Supplementary file 1 [file Data_Sheet_1.ZIP › Supplementary Material Presentation0615/新图片/figure 2.jpg]

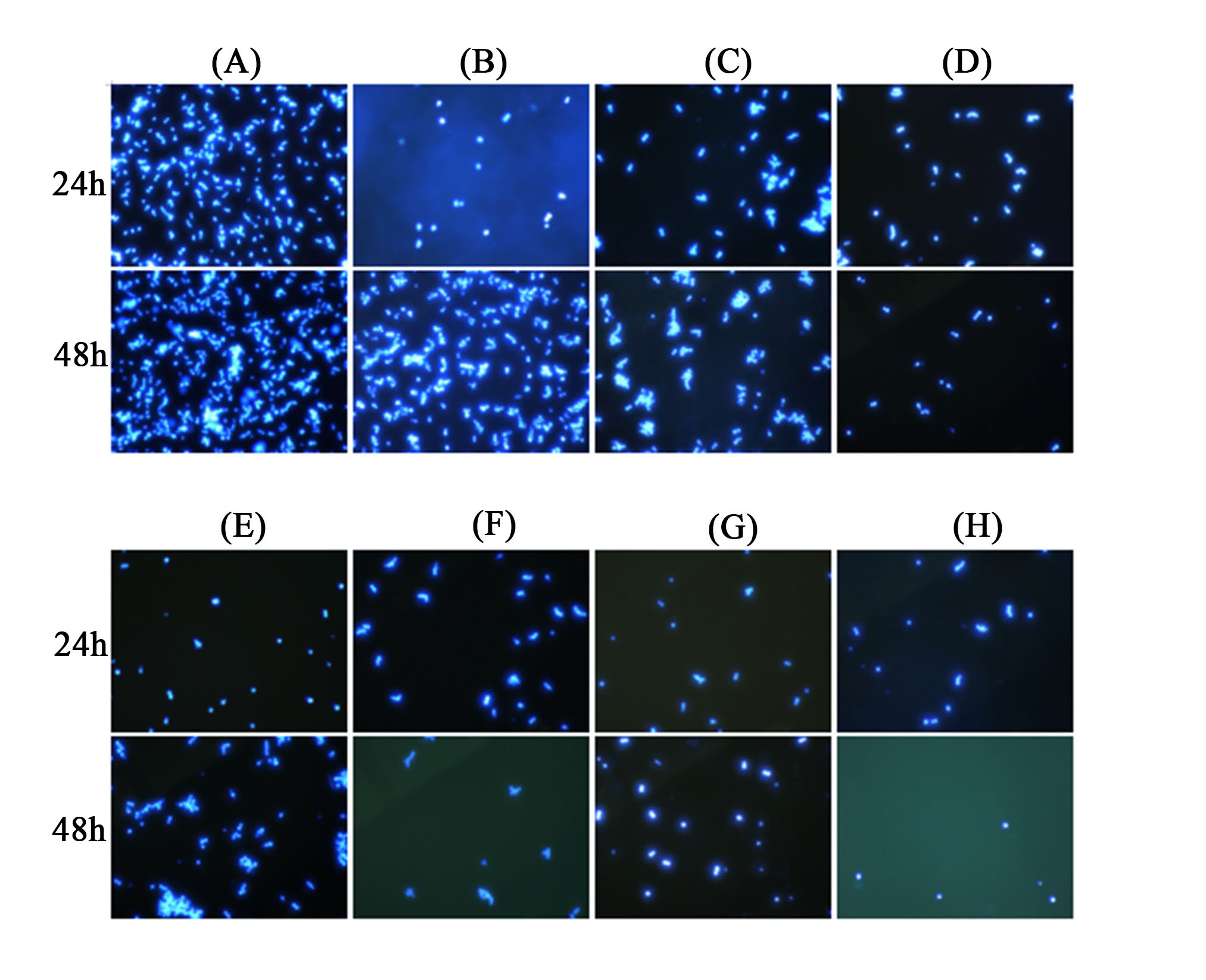

Supplement: Supplementary file 1 [file Data_Sheet_1.ZIP › Supplementary Material Presentation0615/新图片/figure 3.jpg]

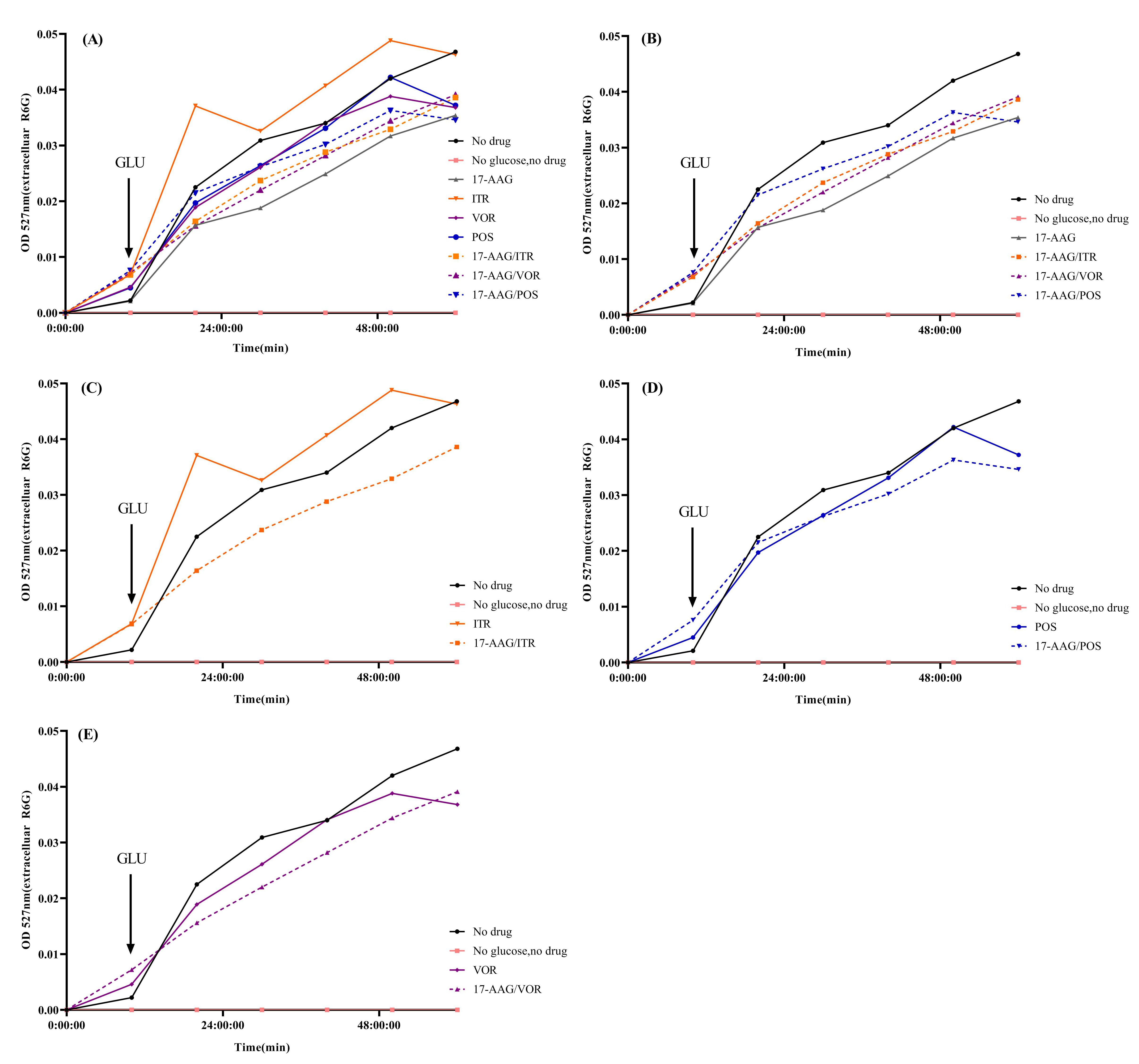

Supplement: Supplementary file 1 [file Data_Sheet_1.ZIP › Supplementary Material Presentation0615/新图片/figure 4.jpg]

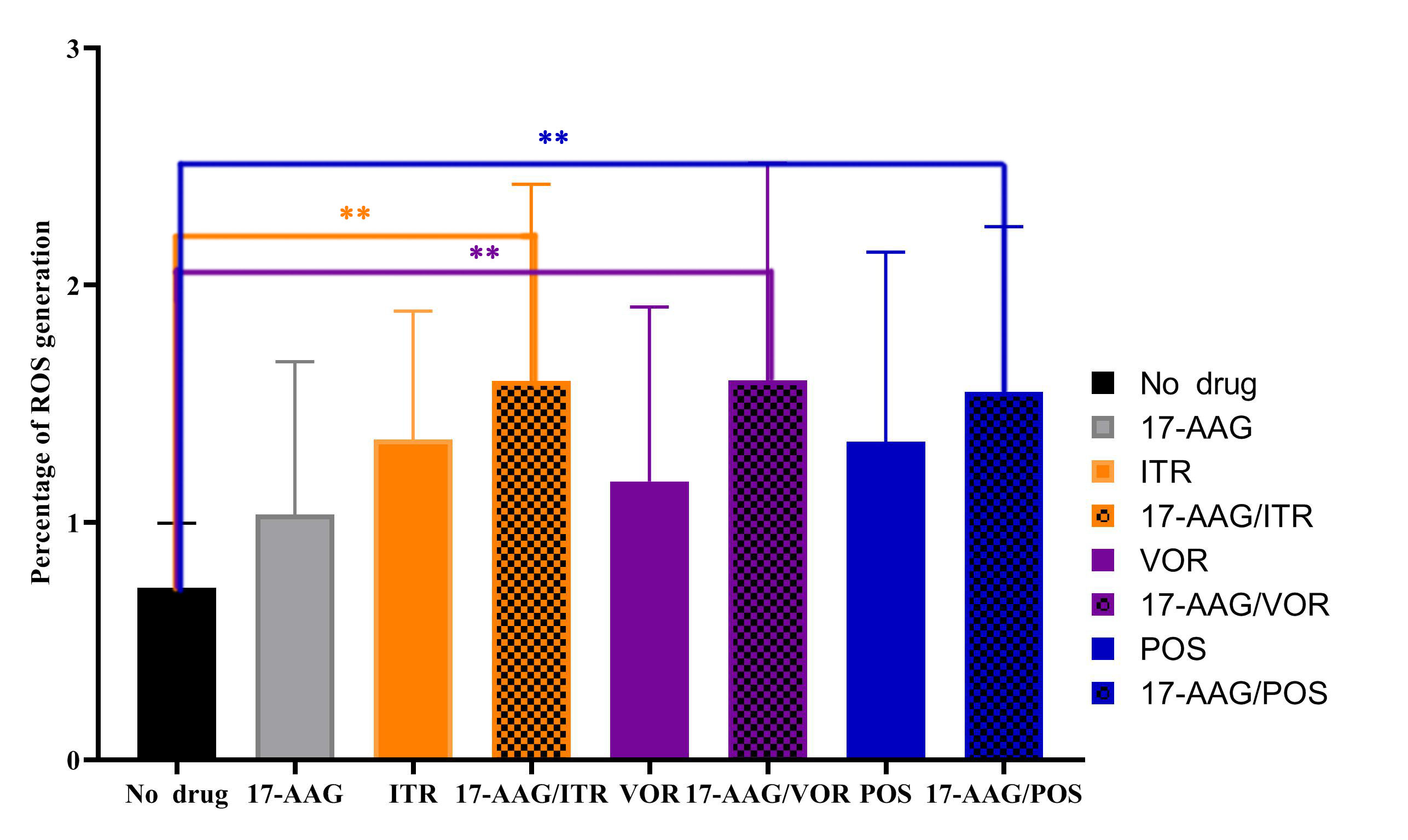

Supplement: Supplementary file 1 [file Data_Sheet_1.ZIP › Supplementary Material Presentation0615/新图片/figure 5.jpg]

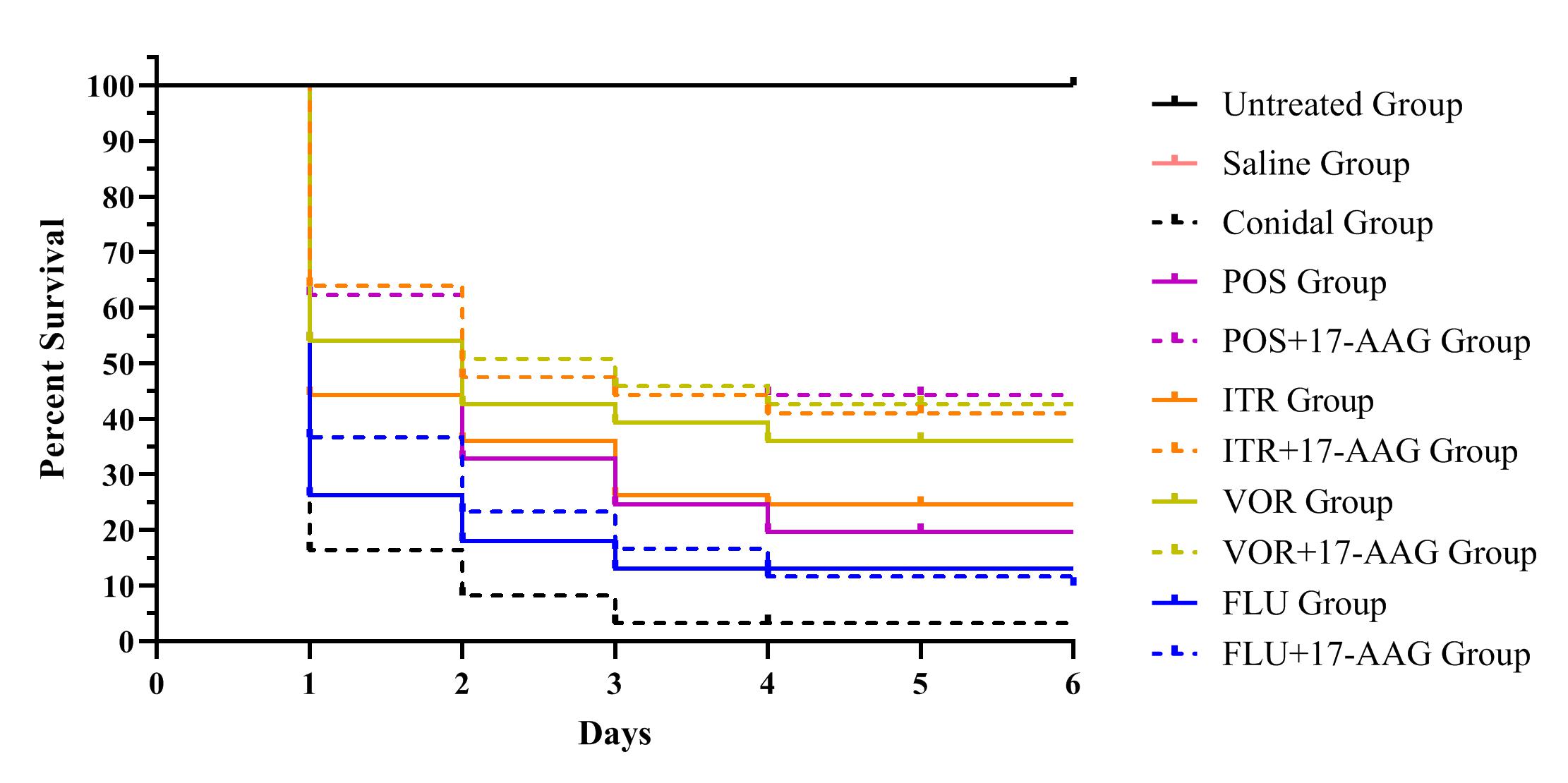

Supplement: Supplementary file 1 [file Data_Sheet_1.ZIP › Supplementary Material Presentation0615/新图片/glabrata B.jpg]

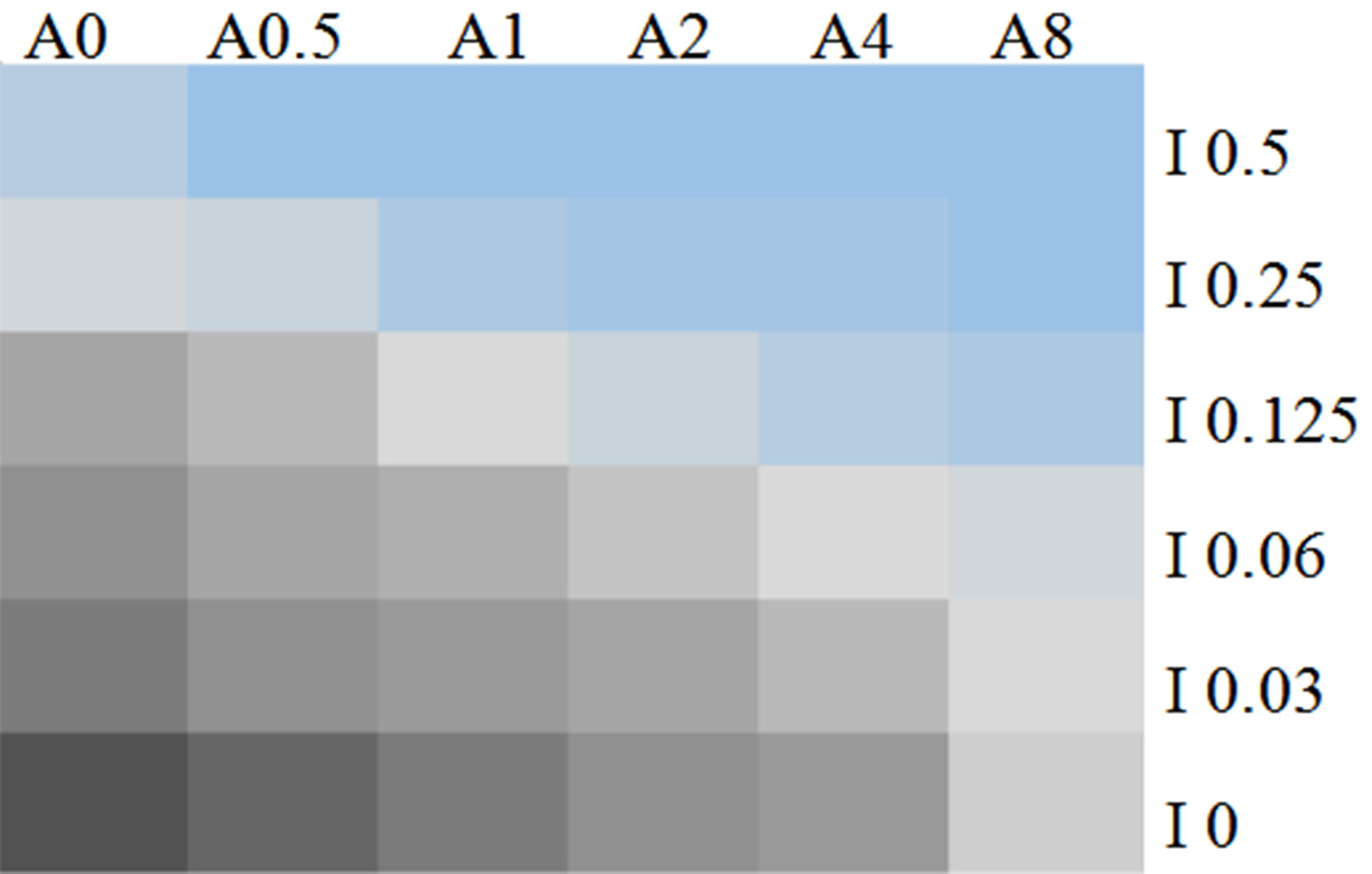

Supplement: Supplementary file 1 [file Data_Sheet_1.ZIP › Supplementary Material Presentation0615/新图片/图片1.jpg]

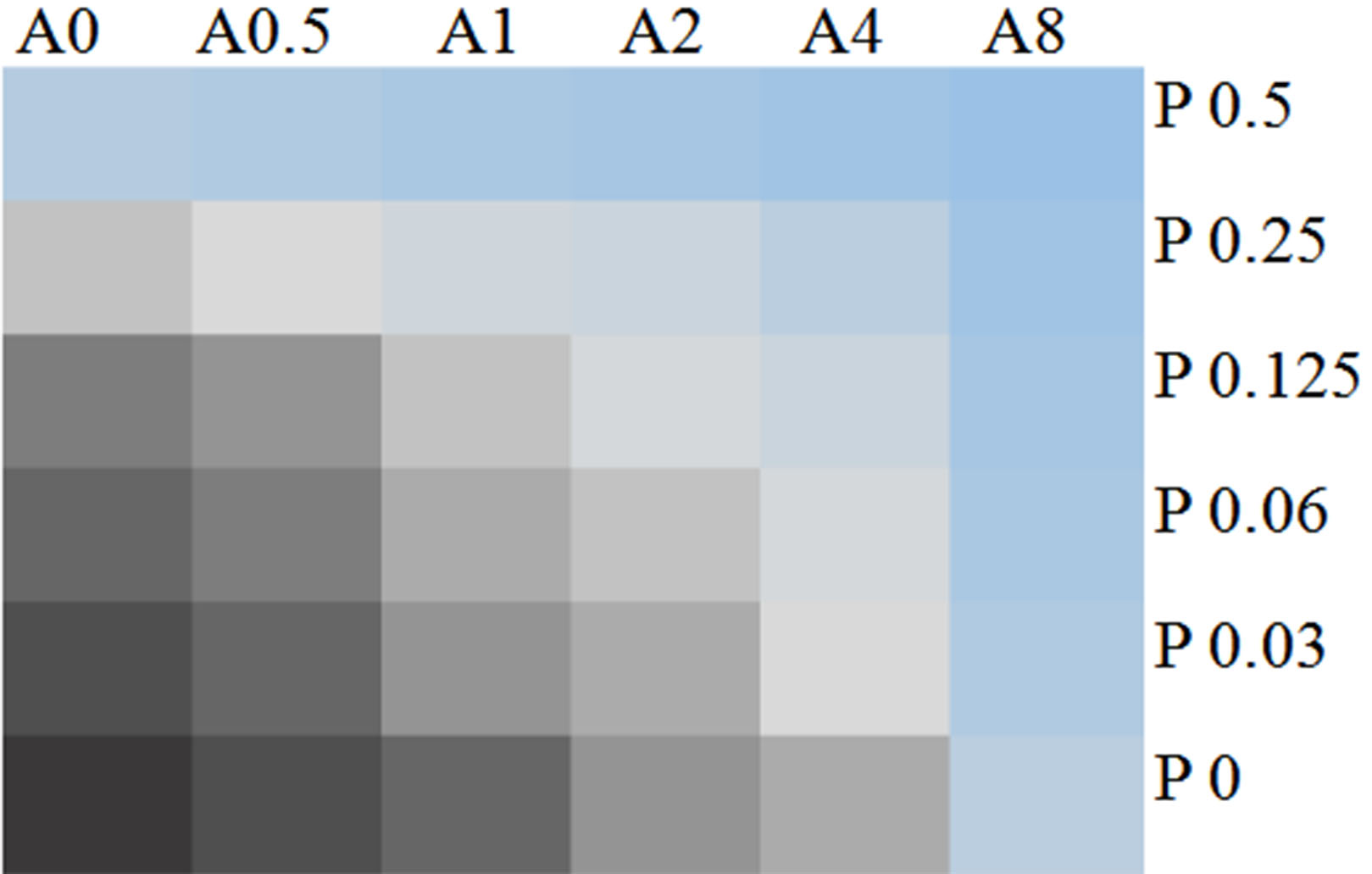

Supplement: Supplementary file 1 [file Data_Sheet_1.ZIP › Supplementary Material Presentation0615/新图片/图片2.jpg]

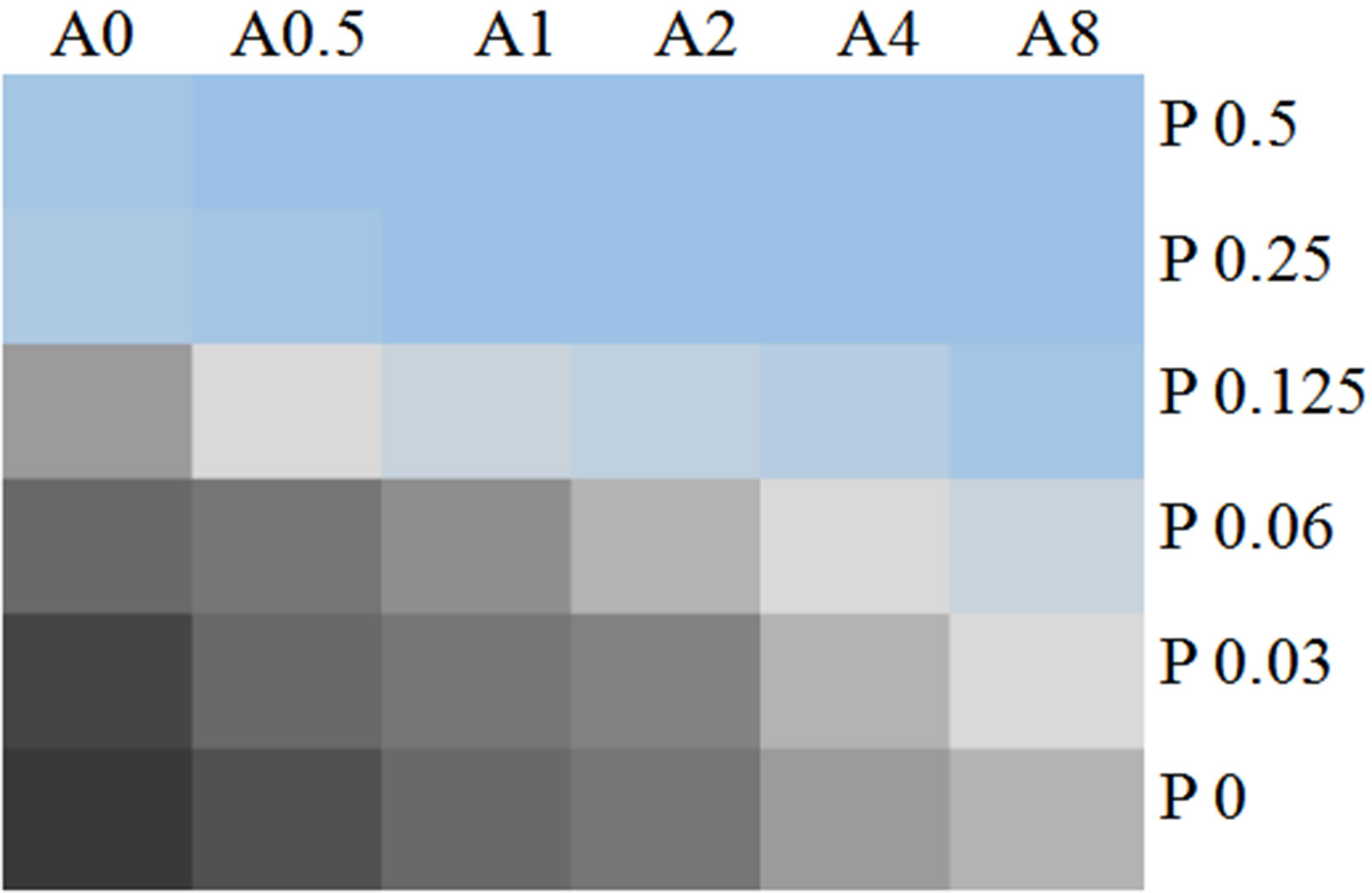

Supplement: Supplementary file 1 [file Data_Sheet_1.ZIP › Supplementary Material Presentation0615/新图片/图片3.jpg]

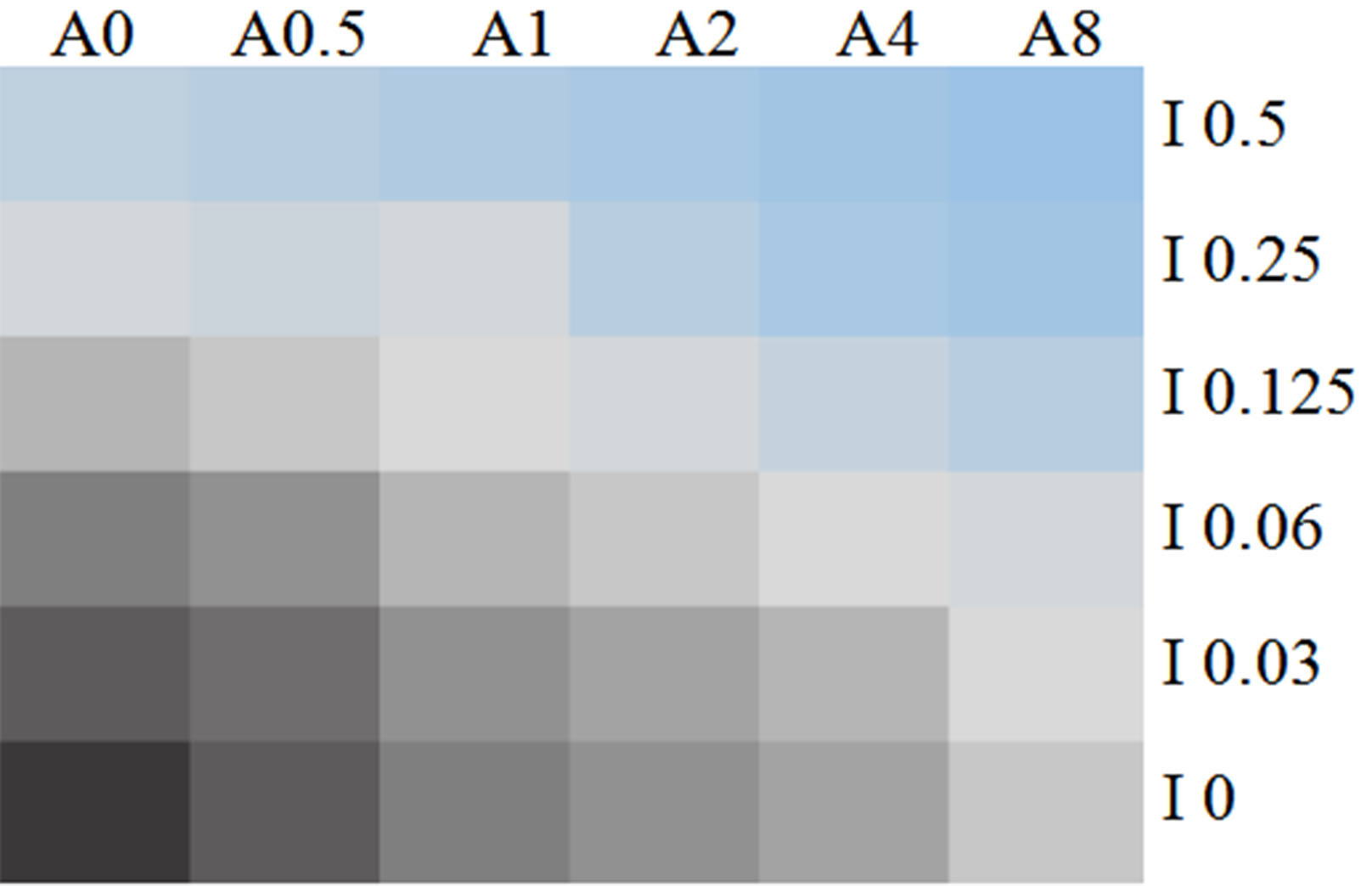

Supplement: Supplementary file 1 [file Data_Sheet_1.ZIP › Supplementary Material Presentation0615/新图片/图片4.jpg]

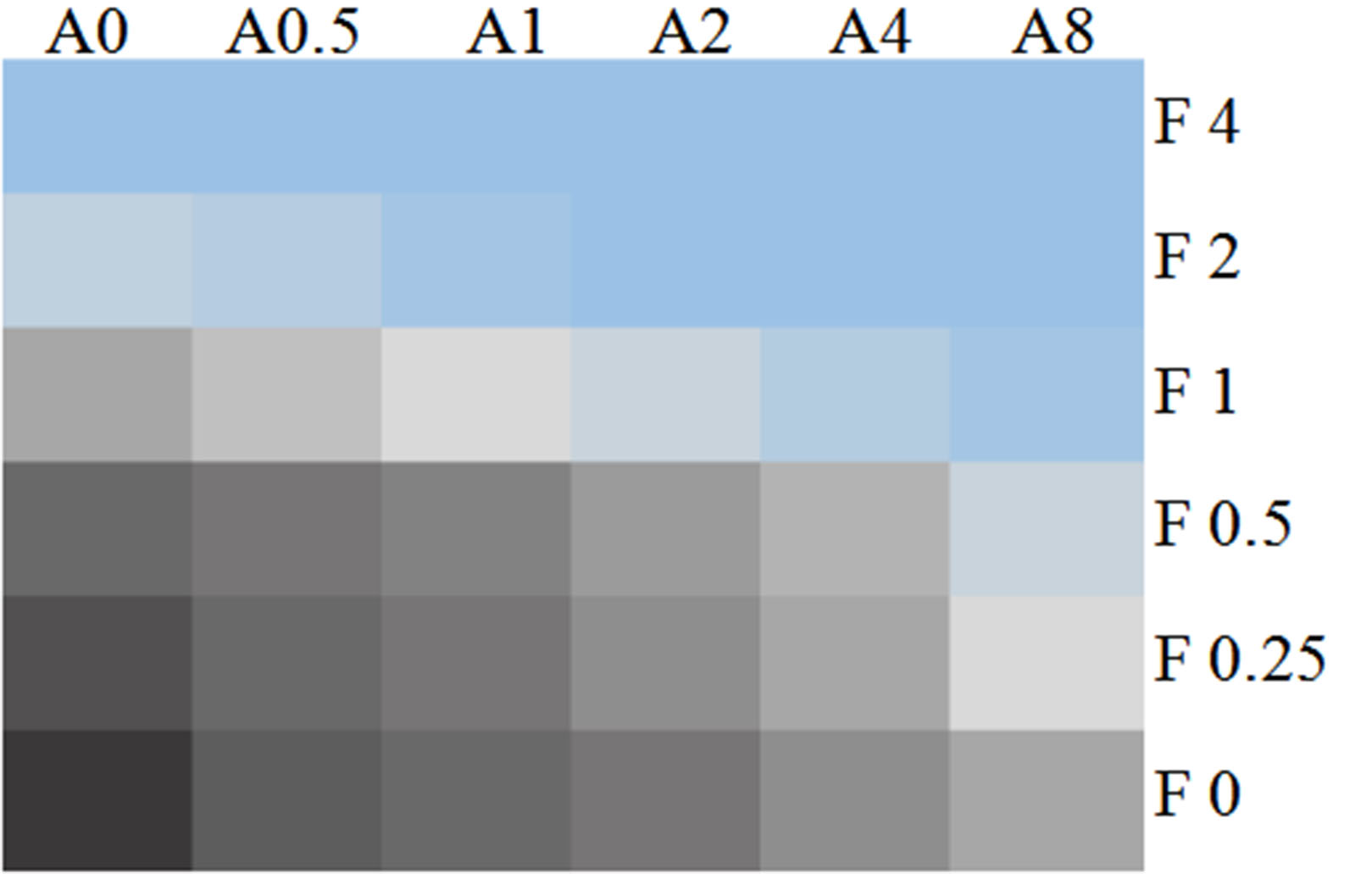

Supplement: Supplementary file 1 [file Data_Sheet_1.ZIP › Supplementary Material Presentation0615/新图片/图片5.jpg]

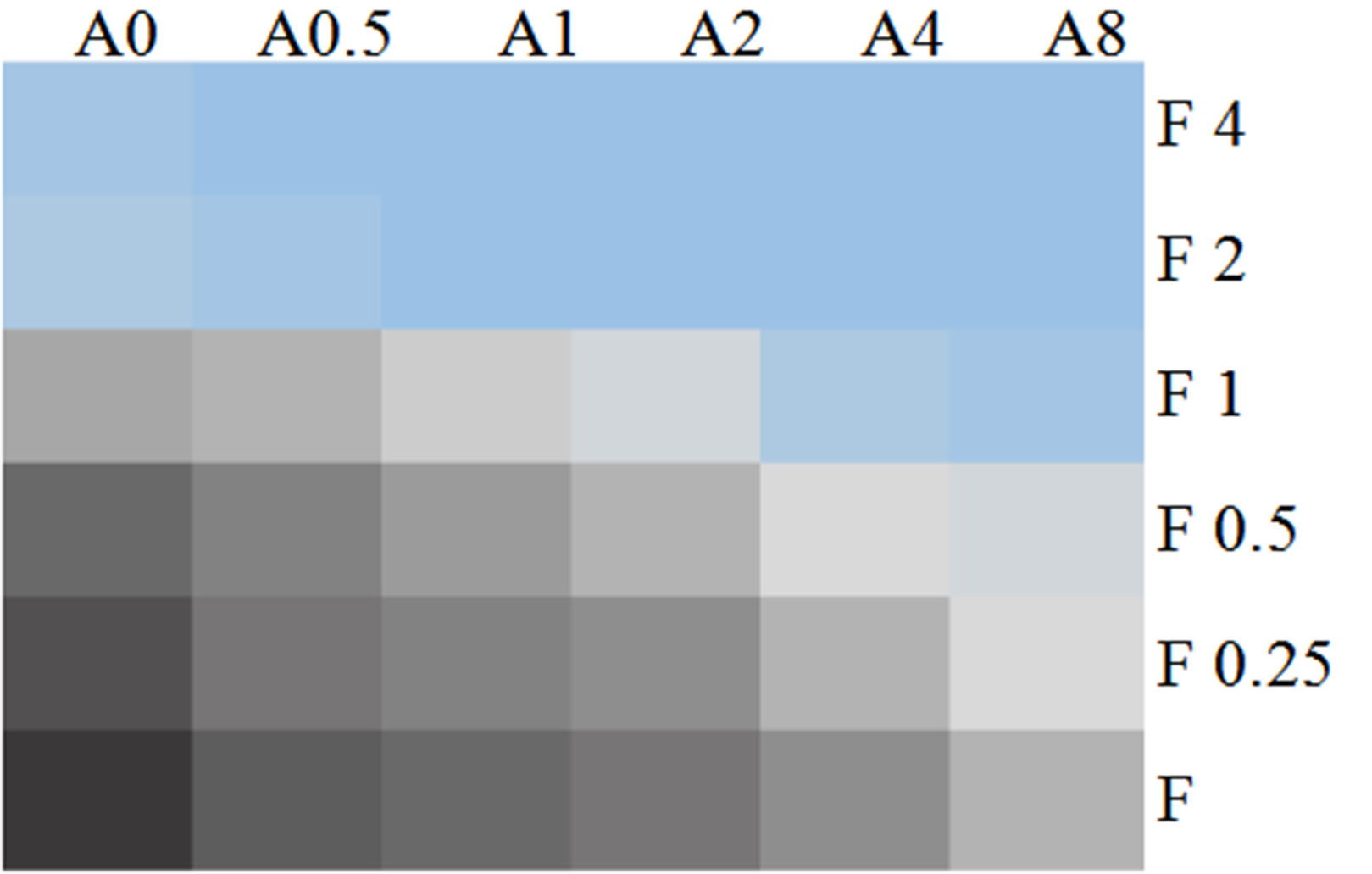

Supplement: Supplementary file 1 [file Data_Sheet_1.ZIP › Supplementary Material Presentation0615/新图片/图片6.jpg]

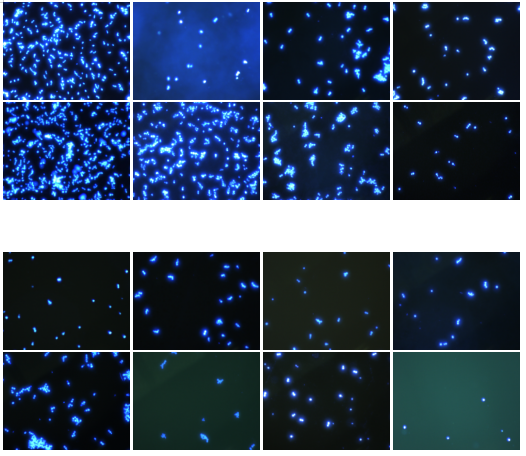

Supplement: Supplementary file 1 [file Data_Sheet_1.ZIP › Supplementary Material Presentation0615/新图片/生物膜.png]
